# Characterization of Natural and Alkaline-Oxidized Proanthocyanidins in Plant Extracts by Ultrahigh-Resolution UHPLC-MS/MS

**DOI:** 10.3390/molecules26071873

**Published:** 2021-03-26

**Authors:** Maarit Karonen, Iqbal Bin Imran, Marica T. Engström, Juha-Pekka Salminen

**Affiliations:** Natural Chemistry Research Group, Department of Chemistry, University of Turku, FI-20014 Turku, Finland; iqbal.imran@utu.fi (I.B.I.); mtengs@utu.fi (M.T.E.); j-p.salminen@utu.fi (J.-P.S.)

**Keywords:** high-resolution mass spectrometry, orbitrap, oxidation, tannins, UHPLC-DAD-MS/MS

## Abstract

In this study, we analyzed the proanthocyanidin (PA) composition of 55 plant extracts before and after alkaline oxidation by ultrahigh-resolution UHPLC-MS/MS. We characterized the natural PA structures in detail and studied the sophisticated changes in the modified PA structures and the typical patterns and models of reactions within different PA classes due to the oxidation. The natural PAs were A- and B-type PCs, PDs and PC/PD mixtures. In addition, we detected galloylated PAs. B-type PCs in different plant extracts were rather stable and showed no or minor modification due to the alkaline oxidation. For some samples, we detected the intramolecular reactions of PCs producing A-type ether linkages. A-type PCs were also rather stable with no or minor modification, but in some plants, the formation of additional ether linkages was detected. PAs containing PD units were more reactive. After alkaline oxidation, these PAs or their oxidation products were no longer detected by MS even though a different type and/or delayed PA hump was still detected by UV at 280 nm. Galloylated PAs were rather stable under alkaline oxidation if they were PC-based, but we detected the intramolecular conversion from B-type to A-type. Galloylated PDs were more reactive and reacted similarly to nongalloylated PDs.

## 1. Introduction

**Figure 1:**
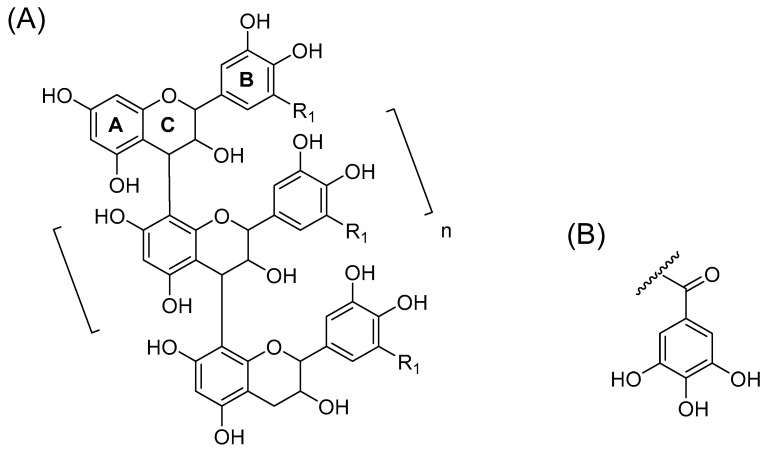
(**A**) A model structure for oligomeric B-type proanthocyanidins (PAs) with C4→C8 linkages: R_1_ = H, procyanidin, R_1_ = OH, prodelphinidin. B-type PAs can also be linked by C4→C6 bonds. A-type PAs have an additional C2→O→C7 or C2→O→C5 ether bond. The hydroxyl groups can also be substituted, for example, galloylated or glycosylated. (**B**) A galloyl group.

Proanthocyanidins (PAs, *syn.* condensed tannins) are oligomers and polymers consisting of flavan-3-ol monomeric units (Figure 1). The diversity of PA structures derives mainly from their hydroxylation patterns, the sequential order of the flavan-3-ol units and the degree of polymerization (DP), in addition to differences in stereochemistry at C2 and C3 and variation in the location and stereochemistry of interflavanoid bonds [1]. The most common PAs are procyanidins (PCs) consisting of (epi)catechin units and prodelphinidins (PDs) consisting of (epi)gallocatechin units (Figure 1). The PA structures presented in the figure have the C4→C8 linkages. However, PAs can also be linked by C4→C6 bonds, and we cannot separate these two linkages from each other by mass spectrometry.

PAs are the most commonly available subgroup of plant tannins, responsible for nearly 90% of the world’s overall market for industrial tannins (>multiple 100-kilo tons annually) and are chemically and economically more appealing as a bio-polymer [2,3,4,5]. Currently, only a few resources, such as the barks or woods of wattle, mimosa, quebracho, oak, chestnut, mangrove, sumach, myrobalans and tara, are used for their production and mainly for the demands of leather tanning, wine, mineral flotation and oil drilling industries, for preparing adhesives, in addition to animal nutrition [4,6,7]. However, even waste or byproducts from handling and processing fruits, vegetables and forest resources from the above listed industrial applications could be a potential source for natural PAs [8,9,10,11]. The PAs could also be further modified in order to enhance their usability and bioactivities [12,13].

The chemical properties of PAs can be modified through derivatization reactions [12]. These reactions include, for example, *O*-acylation by a reaction with acid chlorides or anhydrides or alkylation with alkyl halides. Derivatization modifies the physicochemical properties of PAs for their commercial applications and purification technologies. However, to truly understand the actual reactions happening, several parameters, such as the reagents and solvents used, temperature, pH, and reaction time need to be regulated [12]. A simple and rapid way to improve the usability of plant PAs is to oxidize them to create new PAs with altered molecular structures. Even though the reaction conditions of oxidation are well-known and different reaction mechanisms have been suggested, the corresponding structural changes are mainly known for individual small PAs [14,15,16,17,18]. For example, the oxidation and rearrangement reactions of different monomeric and dimeric flavan-3-ols have been elegantly discussed already 30 years ago, showing that the chemistry of these PAs at alkaline pH is regulated by the formation of A- and/or B-ring quinone-methides as highly reactive intermediates causing the rearrangement reactions and the oxidative conversion of B-type to A-type PAs [15]. Instead, the behavior of PA mixtures in the plant extracts is not known, and this is most probably due to difficulties in the analyzing of structurally different initial PAs, which is challenged even further after oxidation. In our previous study, we tested whether different types of natural PAs could be chemically modified to produce new types of PAs and studied the effects of nonspecific alkaline oxidation mimicking the often-used alkaline extraction process for bark waste [19,20,21,22,23] on 102 PA-containing plant extracts [13]. The results indicated different reactivities for PCs and PDs. The result suggested that plant PAs could be modified at high pH, and the presence of PD groups significantly enhanced the probability of modification reactions. The main reaction route was concluded to be intramolecular, but for PD-rich and galloylated PAs, both intra- and intermolecular reactions were indicated [13]. In addition, other studies have shown that oxidation of PAs includes both intramolecular and intermolecular reactions [14,15,24,25].

The detailed characterization and understanding of natural and oxidized PAs is challenging, but recent advances in ultra-high-performance liquid chromatographic tandem mass spectrometric (UHPLC-MS/MS) instrumentation with high-resolution mass analyzers have enabled the characterization of many different plant PAs [18,26,27,28]. The popular mass analyzers for the characterization of PAs have been orbitrap [27,28] and quadrupole time-of-flight (QTOF) [29,30] because of their high-resolution properties, which allow the determination of the exact masses and corresponding molecular formulae of studied PAs in plant extracts. PAs found in nature usually contain a mixture of different oligo- and polymeric structures, which yields multiply charged ions in electrospray ionization, expanding the mass range used [30,31,32]. In addition, PAs have well-known characteristic fragmentation patterns, which can be used for their identification: these include quinone-methide (QM) cleavage, heterocyclic ring fission (HRF) and retro-Diels–Alder (RDA) fragmentation [27,31,32,33]. The same fragmentation patterns are usually present for both A-type and B-type PAs, and the *m*/*z* values observed for A-type PAs with one ether linkage differ by 2 Da from the corresponding B-type PAs [26,31,32,33,34,35].

In this study, we utilized UHPLC combined with ultrahigh-resolution tandem mass spectrometry in order (a) to study the natural PA structures in 55 PA-rich plant samples in detail, (b) to see the sophisticated changes in the modified PA structures after the aerial oxidation in alkaline conditions and (c) to recognize the typical patterns and models of reactions within different PA classes due to the oxidation.

## 2. Results and Discussion

**Figure 2:**
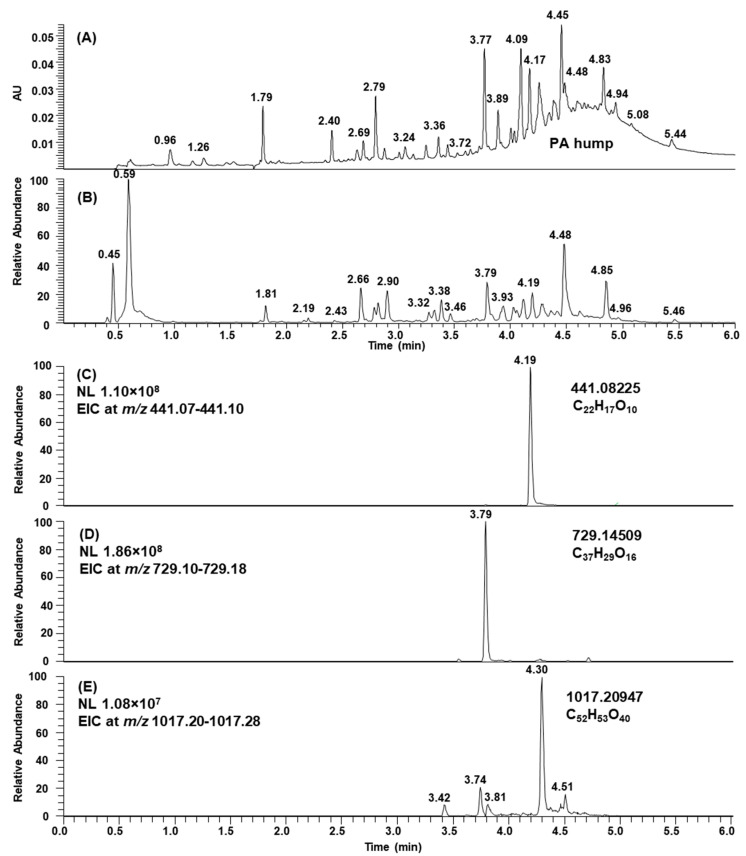
(**A**) UV chromatogram at 280 nm, (**B**) total ion chromatogram, (**C**) extracted ion chromatogram (EIC) at *m*/*z* 441, (**D**) EIC at *m*/*z* 729, and (**E**) EIC at *m*/*z* 1017 of the leaf extract of *Ruprechtia salicifolia*. PA = proanthocyanidin, NL = normalized intensity.

We selected 55 PA-rich plant samples containing interesting but complex mixtures of natural PAs based on our previous study [13]. These plant samples were similarly oxidized by aerial oxidation under alkaline conditions as in [13], and after the oxidation, PAs were even more complex. These natural and modified PAs were studied here by UHPLC-DAD connected to ultrahigh-resolution Q-orbitrap MS/MS in order to detect the sophisticated changes in PA structures. PAs were identified based on their singly and/or multiply charged ions with their corresponding exact masses and molecular formula. The analyses were carried out by reversed-phase LC, and therefore, oligomeric and polymeric PAs were mainly present as unresolved humps in the UV chromatograms at 280 nm, see the leaf extract of *Ruprechtia salicifolia* in Figure 2A for an example. In the total ion chromatogram, the ionization of other phenolic compounds was more intensive than that of the PAs, and therefore, the PA hump was not so apparent (Figure 2B). However, the accuracy of orbitrap makes the interpretation of MS results easier as the isotopic patterns of multiply charged ions are clearly defined, and the possible overlapping of peaks can be detected, thus enabling the determination of the exact masses and molecular formulae of different oligo- and polymeric PAs. For example, galloylated (epi)catechin at *m*/*z* 441, galloylated dimeric PC at *m*/*z* 729, and galloylated trimeric PC at *m*/*z* 1017 exhibit well-separated peaks in extracted ion chromatograms (EICs) in Figure 2C–E, and they can be easily detected based on their UV and mass spectra, including exact masses and the corresponding molecular formula. The results obtained here were consistent with previous results obtained by MS/MS, showing that *Ruprechtia salicifolia* leaves contained 24 mg/g of PAs, of which only 3% were PD-containing PAs, some of them were galloylated, and the mDP was found to be 7 [13].

### 2.1. B-Type PCs in the Initial Non-Oxidized Plant Extracts

B-type PCs in the plant extracts were detected based on their singly and multiply charged molecular ions in addition to the characteristic fragmentation patterns. For example, PCs in *Begonia bowerae* “Nigra” extract exhibited a distinct series of [M‒H]^‒^ ions separated by 288 Da from *m*/*z* 289 to *m*/*z* 1729 corresponding for B-type PCs from monomer to hexamer and of [M‒2H]^2‒^ ions separated by 144 Da from *m*/*z* 1008 to *m*/*z* 1296 corresponding for B-type PCs from heptamers to nonamers (Table S1). The mass spectra of monomeric PCs, i.e., flavan-3-ols catechin and epicatechin, exhibited a characteristic fragment ion at *m*/*z* 245 as reported previously [31,32,36].

**Figure 3:**
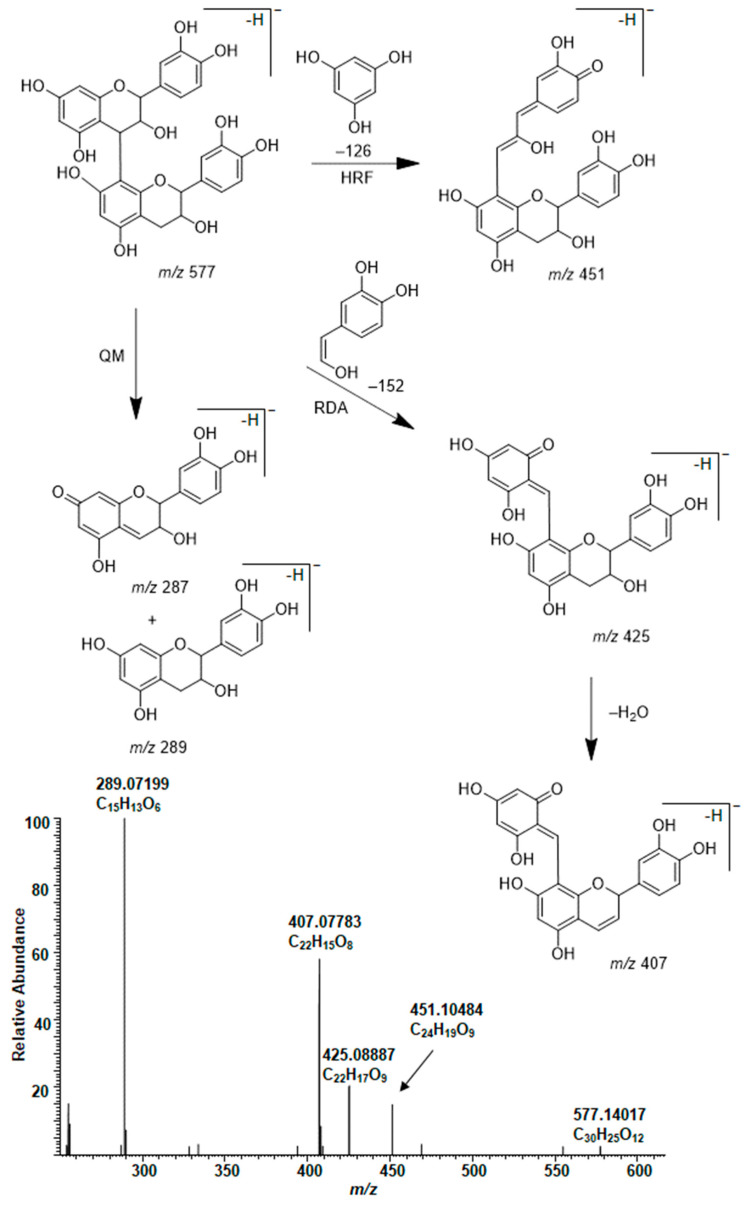
Characteristic fragmentation pathway and the MS/MS of B-type procyanidin dimer in the leaf extract of *Cunninghamia lanceolata*. The mechanisms are heterocyclic ring fission (HRF), retro-Diels‒Alder (RDA) fragmentation and quinone methide (QM) cleavage [31,32,33].

In general, the MS/MS of B-type PC dimers showed the characteristic fragmentation patterns yielding ions at *m*/*z* 287 and 289 (QM cleavage), *m*/*z* 425 and 407 (RDA fragmentation and the sequential water elimination) and *m*/*z* 451 (HRF; Figure 3). The RDA fragmentation is considered to be the most important fragmentation pattern for the characterization of B-type PC dimers, and the fragmentation on the extension unit has been thought to be energetically more favorable as it produces fragment ions with larger π–π hyperconjugated system than the RDA fragmentation on the terminal unit [31,32,33].

### 2.2. A-Type PCs in the Initial Non-Oxidized Plant Extracts

**Figure 4:**
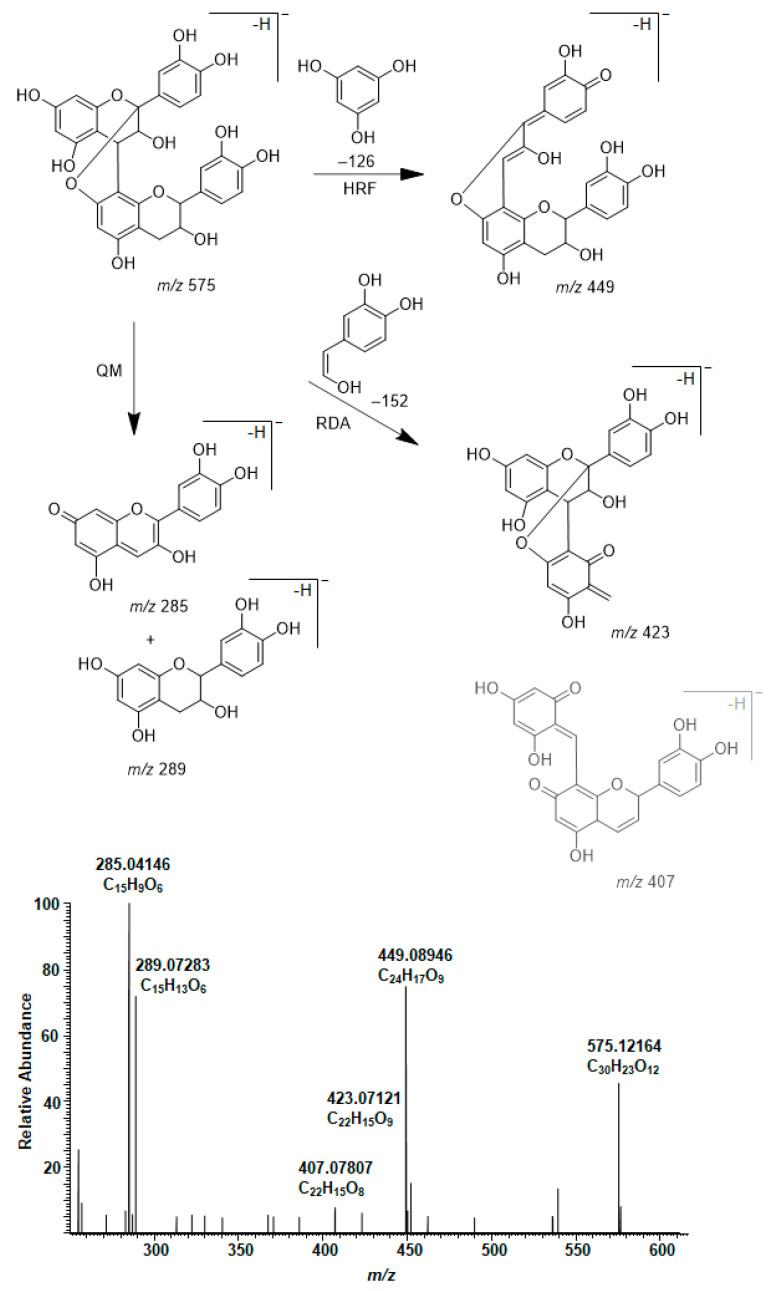
Characteristic fragmentation pathway and the MS/MS of A-type procyanidin dimer in the leaf extract of *Aglaonema crispum*. The mechanisms are heterocyclic ring fission (HRF), retro-Diels–Alder (RDA) fragmentation and quinone methide (QM) cleavage [26,34]. The ion at *m*/*z* 407 is a tentative suggestion for the RDA fragmentation and the sequential water elimination of the extension unit.

**Figure 5:**
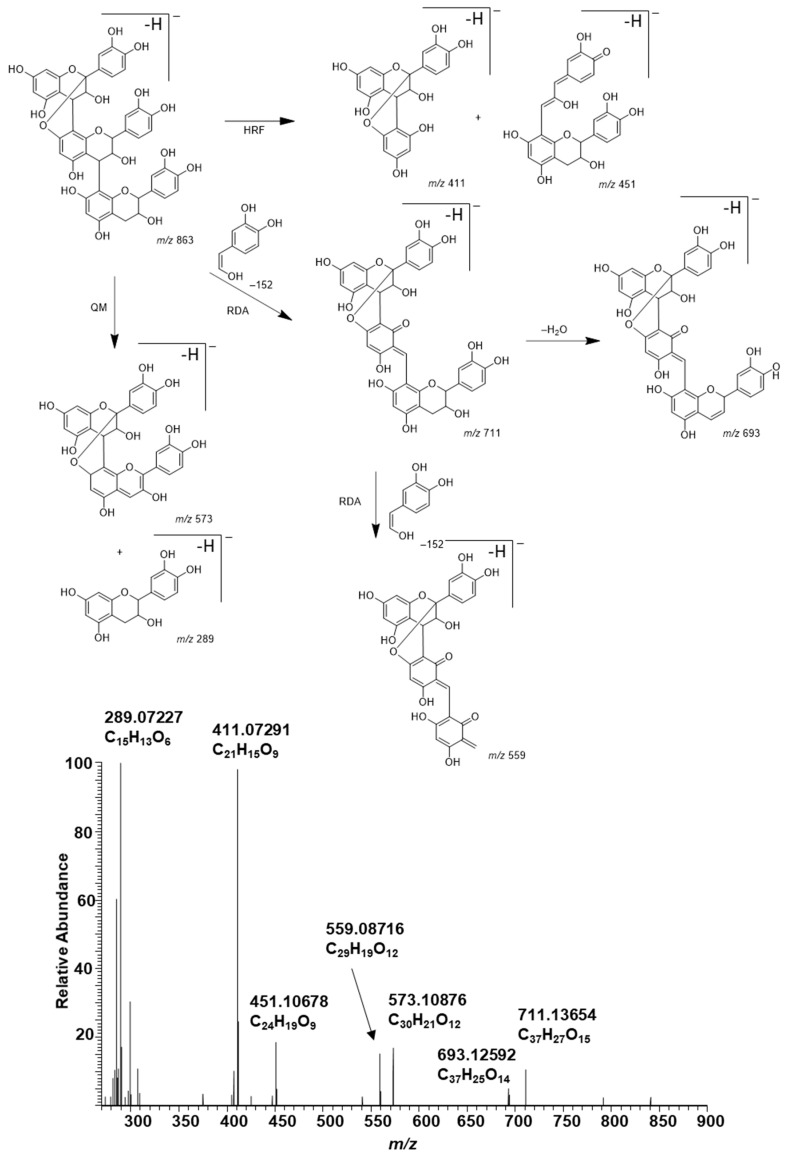
Characteristic fragmentation pathway and the MS/MS of A-type procyanidin trimer in the leaflet extract of *Tectaria macrodonta*. The mechanisms suggested are heterocyclic ring fission (HRF), retro-Diels‒Alder (RDA) fragmentation and quinone methide (QM) cleavage.

The presence of A-type PAs in the initial plant extracts was confirmed by their characteristic fragmentation patterns similarly to B-type PAs. As examples, the characteristic fragmentation pathways and the MS/MS data of A-type PC dimer and trimer having one A-type linkage are shown and discussed in detail (Figure 4 and Figure 5). The fragmentation of the A-type PC dimer produced four fragment ions (Figure 4). The RDA fragmentation of the terminal unit produced an ion at *m*/*z* 423, which confirms the presence of an A-type linkage [26,34,35]. In addition, we suggest that the RDA fragmentation of the extension unit and the sequential loss of water could produce the ion at *m*/*z* 407. The ion at *m*/*z* 449 is the HRF production, i.e., the result of the loss of the phloroglucinol unit [26]. The ions at *m*/*z* 285 and 289 corresponded to QM cleavage. Similarly, the A-type PC trimer exhibited a molecular ion at *m*/*z* 863 and characteristic fragment ions in MS/MS at *m*/*z* 711, 693, 573, 559, 451, 411 and 289 as previously reported by Sui et al. (2016) [26]. The RDA fragmentation of the A-type PC trimer produced an ion at *m*/*z* 711, which again confirms the presence of A-type linkage according to the previous study [26] and hints that the A-type linkage could be between two extension units. We suggest that the ion at *m*/*z* 693 corresponds to the sequential loss of water and supports the position of A-type linkage, as shown in Figure 5. In addition, we suggest that the additional RDA of the heterocyclic C-ring of the terminal flavan-3-ol unit exhibits the ion at *m*/*z* 559. This ion was minor in our studies. The ions at *m*/*z* 573 and 289 corresponded to the QM cleavage of the lower interflavanoid bond [26]. We also detected ions at *m*/*z* 575 and 287. We propose that the ions at *m*/*z* 451 and 411 could correspond to the HRF of the heterocyclic C-ring of the middle flavan-3-ol unit supporting the location of A-type linkage between the extension units according to Figure 5.

### 2.3. Modifications of B-Type PCs in Plant Extracts Due to the Alkaline Oxidation

**Figure 6:**
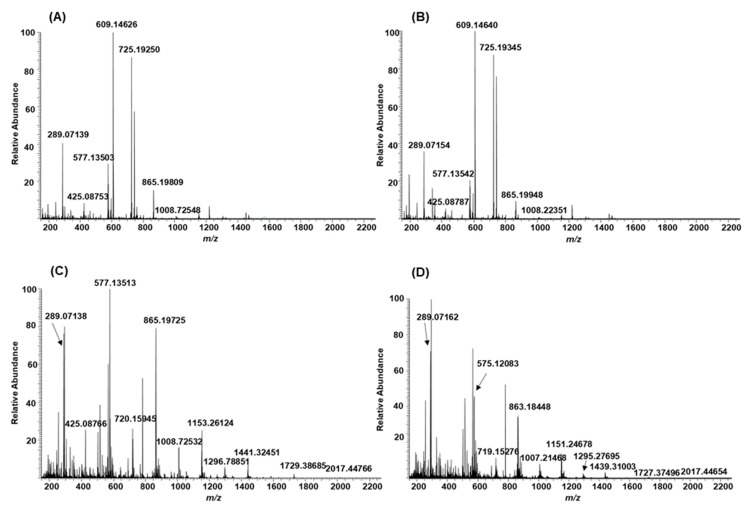
Total mass spectra of non-oxidized (**A**) and oxidized (**B**) leaf extract of *Begonia bowerae* “Nigra” and of non-oxidized (**C**) and oxidized (**D**) leaflet extract of *Cyperus owanii*. The exact masses of main ions of proanthocyanidins are listed in Tables S1 and S2.

After the oxidation, B-type PCs in different plant extracts were modified differently. Some of the samples showed no or minor modifications; see Figure 6A,B, for example, for shorter B-type PC oligomers in the leaf extract of *Begonia bowerae* “Nigra” before and after oxidation. When these types of plant extracts were oxidized, the total mass spectra of the initial and oxidized plant extracts were similar. However, tiny differences were detected in the detailed mass spectrometric data. For example, the PC dimer at *m*/*z* 577 was companioned with *m*/*z* 575, and the PC trimer at *m*/*z* 865 with *m*/*z* 863, respectively. When the amounts of PCs were low before the oxidation, after the oxidation their signals almost disappeared from the mass spectra, hinting that they had converted to unidentifiable or degraded due to the oxidation. This phenomenon was observed, for example, for the leaf extracts of *Combretum indicum* and *Euphorbia characias* containing low amounts of few short PC oligomers and supported by the previous MS/MS data that showed that the PA content decreased from 4 mg/mL to 1 mg/mL and 7 mg/mL to 1 mg/mL, respectively, due to the oxidation [13]. This phenomenon might be related to the experimental conditions used, meaning that they were more severe when the initial PA contents were low. In some samples, a part of the B-type PCs was modified more, see Figure 6C,D, for example, for shorter B-type PC oligomers in the leaflet extract of *Cyperus owanii* before and after oxidation. When these types of plant extracts were oxidized, visible differences were detected in the total mass spectra showing the mass difference of 2 Da in comparison to initial PCs (Figure 6C,D, Table S2).

**Figure 7:**
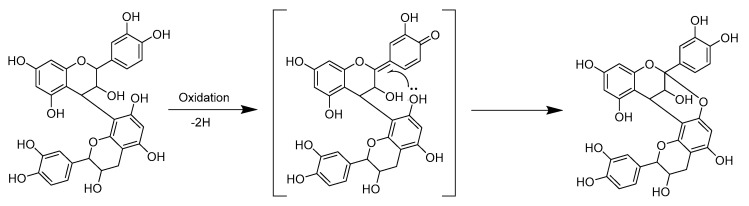
The suggested mechanism for the conversion of B-type procyanidin dimer to A-type dimer according to [15,37].

It is well-known that the oxidation of *o*-dihydroxy polyphenols, i.e., the catechol B-ring of PCs, typically yields *o*-quinones with a mass difference of 2 Da. In fact, we have noticed that this kind of oxidation can also happen in mass spectrometric analyses. There can be minor signals in the mass spectra having the *m*/*z* values 2 Da smaller than the *m*/*z* values of PCs corresponding for the possible oxidation or formation of quinone forms of PCs during the ionization (data not published). It is feasible that here in the alkaline oxidation, *o*-dihydroxy polyphenols form first *o*-quinones. However, the *o*-quinone forms formed are unstable and most probably quickly react further. One possible outcome could be the oxidative conversion of B-type PCs to A-type PCs, as previously reported in the literature [15]. The transformation of B-type PCs to A-type PCs involves the oxidative removal of the hydride ion at C2 of the C-ring as an initial step: the prevailing basic conditions induce the oxidation of the *o*-dihydroxy functionality of B-ring to an *o*-quinone, which subsequently serves as an oxidant for the conversion of B-type procyanidin dimer to A-type one [15] (Figure 7). This quinone methide mechanism has also been evidenced in different temperatures, pH and catalytic conditions [37], by radical oxidation using 1,1-diphenyl-2-picrylhydrazyl radicals under neutral conditions [38] and by laccase (EC 1.10.3.2) [39].

**Figure 8:**
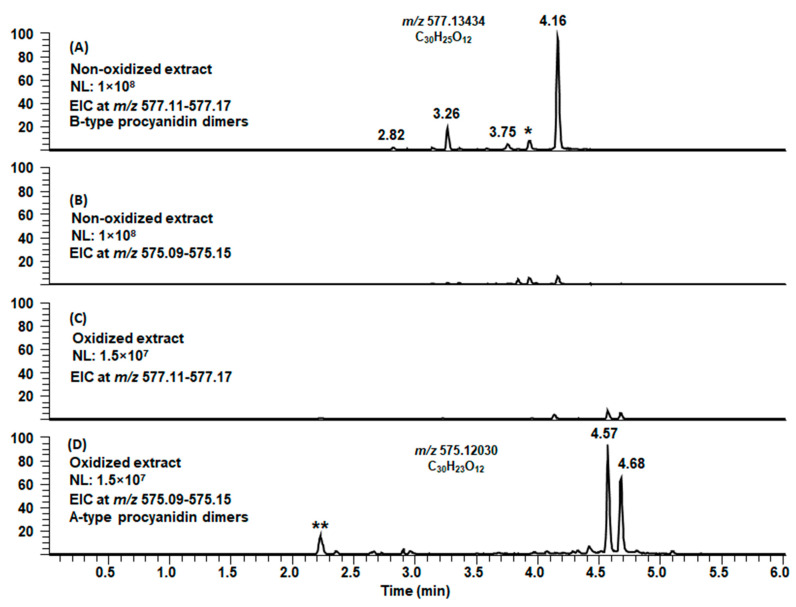
Extracted ion chromatograms (EICs) of the non-oxidized leaflet extract of *Microgramma mauritiana* (**A**) the ions at *m*/*z* 577.11–577.17 showing the presence of B-type procyanidin dimers, (**B**) the ions at *m*/*z* 575.09–575.15 and of the oxidized extract of *Microgramma mauritiana* (**C**) the ions at *m*/*z* 577.11–577.17 and (**D**) the ions at *m*/*z* 575.09–575.15 showing the presence of A-type procyanidin dimers. (*) the ion corresponds to the isotopic signal of a doubly charged molecular ion for a tetrameric procyanidin. (**) the ion has the same elemental composition as A-type PC dimers but different fragment ions, NL = normalized intensity. Note that this study was qualitative, and the intensities cannot be compared as such.

When we studied the modified PCs with the mass difference of 2 Da in the oxidized extracts and their product ions obtained by MS/MS, they were concluded to be similar to A-type PCs and their fragment ions (as discussed above in Section 2.2), showing the characteristic fragmentation patterns yielding ions at *m*/*z* 285 and 289 (QM cleavage), *m*/*z* 423 (RDA fragmentation) and *m*/*z* 449 (HRF). As an example, we show the conversion of the B-type PC dimers of leaflet extract of *Microgramma mauritiana* to A-type PC dimers in Figure 8. In the non-oxidized extract, we detected four B-type PC dimers with *m*/*z* values 577 (Figure 8A) but no A-type PC dimers with *m*/*z* values 575 (Figure 8B). The tiny peaks in the EIC at *m*/*z* 575 corresponded presumably for the oxidation of B-type PCs in the ion source as the retention times are exactly the same as for B-type PCs. In the oxidized extract, we detected only traces of the initial B-type PCs at *m*/*z* 577 (Figure 8C), but instead, we detected intensive peaks at later retention times corresponding for A-type PCs at *m*/*z* 575 (Figure 8D). The tiny peaks in the EIC at *m*/*z* 577 having the very same retention times corresponded for the isotopic signals of the *m*/*z* 575. It must be noted that this study was qualitative, meaning that the abundances of the ions cannot be compared as such. In addition, it must be noted that the conversion of B-type PC to A-type PC was not always as complete as in this example and that all B-type PCs were not converted to A-type PCs; roughly estimated the abundances of the ions of A-type and B-type PC oligomers were equal. On the other hand, in some samples, we detected more differences of 2 Da; for example, the PC heptamer at *m*/*z* 2017 could be companioned with ions at *m*/*z* 2015 and 2013, hinting at the formation of one and two A-type linkages, respectively.

Even though the conversion of B-type PCs to A-type seemed to be the main reaction mechanism, we cannot fully exclude other modification reactions. PAs were present as complex mixtures in the initial non-oxidized extracts, and these mixtures were even more complex after the alkaline oxidation producing an enormous amount of MS data. For example, previous studies on base-catalyzed oxidation and rearrangement reactions of PAs have shown that PC dimers can converse into different products of C-ring isomerization, including tetrahydroxypyranochromenes, also known as phlobatannins [15]. These products would exhibit very similar *m*/*z* values at 577 but different retention times in LC and different fragmentation patterns in MS. Based on the EICs at *m*/*z* 577, we did not detect rearrangements of this kind in the reaction conditions used. The rearrangements and the opening of the heterocyclic C-ring could also produce other products via regio-isomerization, epimerization or 1,3-aryl migration [15]. The epimerization has mainly been related to manufacturing processes and, for example, the high temperatures related to the roasting of cocoa leads to flavan-3-ol losses, but also to the epimerization of flavan-3-ol monomers, dimers and trimers [40]. In addition, it has been reported that polyphenol oxidase in banana fruit flesh caused the epimerization of (-)-epigallocatechin to (-)-gallocatechin [41]. We looked at the possible epimerization of flavan-3-ols using EICs at *m*/*z* 289, and in general, we did not detect significant epimerization caused by the alkaline oxidation used. However, we cannot fully exclude the epimerization as we detected higher amounts of (+)-catechin in relation to (−)-epicatechin in the oxidized extract of *Pavonia cauliflora* flowers than in the initial non-oxidized extract (Figure S1). It must also be noted that the oxidized extracts were neutralized prior to the UHPLC-MS/MS, which may have caused redox reactions. In the oxidized forms, quinones are electrophiles that can react with the nucleophilic water present, and this could simply result in the reduction reaction yielding B-type PCs, which could also explain why some of the B-type PCs seemed to be unaffected.

### 2.4. Modifications of A-Type PCs in Plant Extracts due to the Alkaline Oxidation

**Figure 9:**
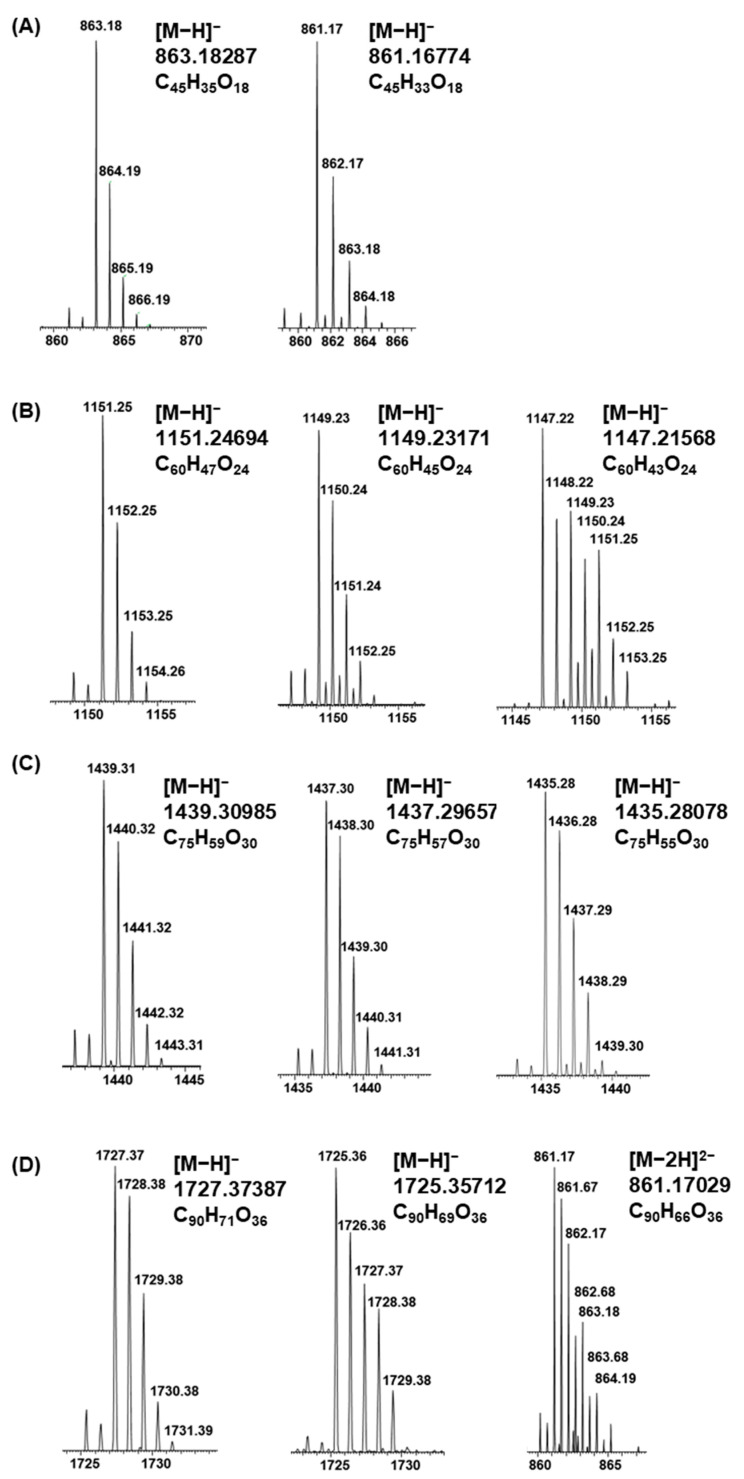
Molecular ions with corresponding exact masses and molecular formulae for procyanidin oligomers with one or more A-type linkages from the leaflet extract of *Tectaria macrodonta*: (**A**) trimers, (**B**) tetramers, (**C**) pentamers, and (**D**) hexamers. The first ion of each oligomer (having one ether bond) is taken from the non-oxidized extract, and the other ions of the oligomer (having two or more ether bonds) from the oxidized extract. For A-type procyanidin hexamer with three ether bonds, the doubly charged ion was more abundant.

A-type PCs in different plant extracts reacted differently due to the alkaline oxidation. Some of the samples showed no or minor modifications. When these types of plant extracts were subjected to alkaline oxidation, no significant differences were detected in the total mass spectra (for example, see Table S3 for the major ions observed in the leaf extract of *Aglaonema commutatum* var. *maculatum* before and after oxidation). However, tiny differences could be detected in the detailed mass spectrometric data having the mass difference of 2 Da and hinting that additional A-type ether linkages could be formed. This phenomenon was more evident for some of the samples with A-type PCs; these A-type PCs were noticed to form additional A-type ether linkages due to the alkaline oxidation (Figure 9). When these types of plant extracts were oxidized, distinct differences were detected in the total mass spectra showing the mass difference of 2 Da in comparison to initial A-type PCs. The [M − H]^−^ ion at *m*/*z* 863 corresponded for a PC trimer having one A-type ether bond and the ion at *m*/*z* 861 for a PC trimer having two ether linkages (Figure 9A). Similarly, tetrameric PCs having one, two and three ether linkages were detected by the [M − H]^−^ ions at *m*/*z* 1151, 1149 and 1147, respectively (Figure 9B). In proportion, pentameric and hexameric PCs having one, two and three ether bonds were detected by the [M − H]^−^ ions at *m*/*z* 1439, 1437 and 1435 and at *m*/*z* 1727, 1725 and 1723, respectively (Figure 9C,D; however, the [M − 2H] ^2−^ ion at *m*/*z* 861 of a PC hexamer was explicitly more abundant than the [M − H]^−^ ion at *m*/*z* 1723 and therefore, shown in the figure).

The formation of additional A-type linkages also affected the retention times of these oligomers. The number of additional ether linkages seemed to some extent increase as the degrees of polymerization of PCs increased, which is expected as there are more positions for the additional bonds. However, it must also be noticed that there can be minor signals with the mass difference of 2 Da corresponding to the possible oxidation or formation of quinone forms of PCs during the ionization, as discussed above. These peaks are also visible in the mass spectra in Figure 9 before the main peaks corresponding for A-type PCs. The abundances of the ions, the shapes of the isotopic patterns and the characteristic fragment ions obtained by MS/MS confirmed the observation of additional A-type ether linkages. It is also important to note that these intramolecular reactions observed did not seem to affect the mean degree of polymerization as the higher PA oligomers or polymers were not detected in the oxidized extracts in comparison to non-oxidized extracts. The observation is supported by Mouls and Fulcrand (2012) [14].

### 2.5. Plant Extracts with PAs Having Both PC and PD Subunits and Their Modifications Due to the Alkaline Oxidation

**Figure 10:**
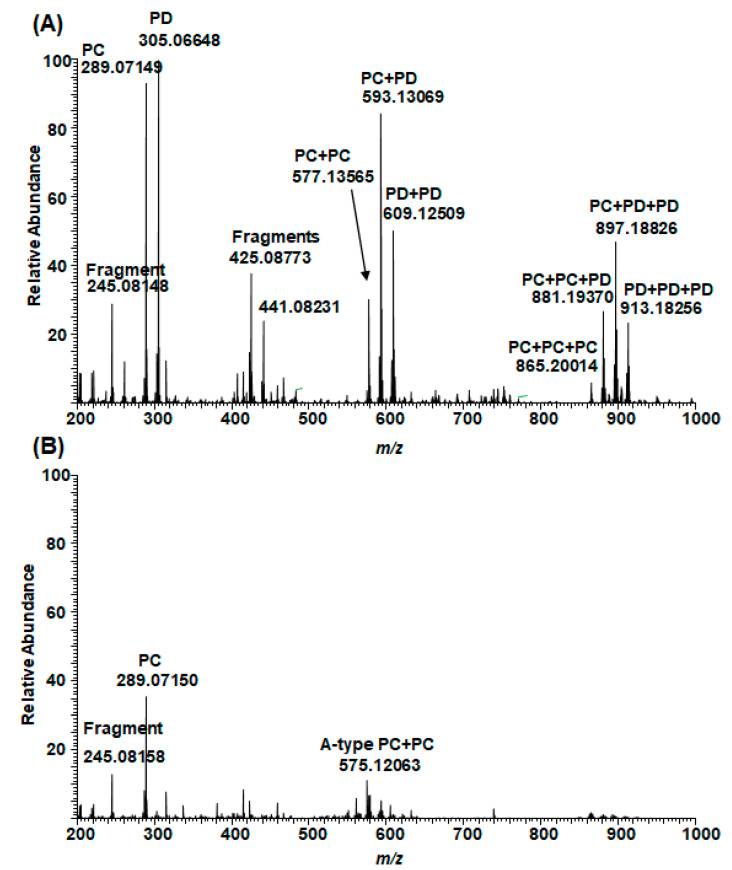
The mass spectra of small mixed B-type oligomeric procyanidins (PCs) and prodelphinidins (PDs) in the non-oxidized (**A**) and oxidized (**B**) leaf extract of *Podocarpus macrophyllus*. The exact masses of main ions are listed in Table S4. The abundances of the ions are fixed to normalized intensity of 6.9 × 10^6^.

Many of the samples contained PAs with both (epi)catechin and (epi)gallocatechin units, i.e., the PAs were PC/PD mixtures. The fate of these PC/PD mixtures due to the modification via alkaline oxidation was different in comparison to PCs. PC/PD mixtures were clearly detected in the plant extracts before the oxidation both by UV and MS; see, for example, the small PA oligomers in the initial leaf extract of *Podocarpus macrophyllus* (Figure 10A and Table S4). After the oxidation, the modified PAs were still detected by UV at 280 nm as a different type and/or delayed hump (Figure S2), but they were not detected anymore by MS, see, for example, the missing PA oligomers in the oxidized leaf extract of *Podocarpus macrophyllus* (Figure 10B and Table S4). We did neither detect any other signals that could correspond to the modified PC/PD mixtures nor to their possible degradation products. For example, for the PC dimer at *m*/*z* 577, we could detect the corresponding A-type PC dimer at *m*/*z* 575 in the oxidized extract, but similar distinct observations could not be made for PC/PD dimers or trimers (Figure 10B). The reason for the loss of detectable PC/PD oligomers in ESI-MS can be, for example, intermolecular reactions between PAs [13,14,15,24,25]. The intermolecular reactions are known to result in the formation of modified PAs by the connection of two distinct oligomeric chains, and typically, they lead to an increase in the mean degree of polymerization [14]. For example, Vernhet et al. (2014) have noticed by small-angle X-ray scattering experiments that if PAs are oxidized in concentrated solutions, the modified PAs are high polymers with long linear or branched chains [25]. Further oxidation can also lead to cyclization between A and B rings of different PAs [24]. In addition, it has been noted that these new bonds and structures are resistant to acid-catalyzed cleavage, and therefore, the increase in the mean degree of polymerization can be estimated only by non-depolymerizing methods [14,24]. However, our results showed that these modified PAs are not either detected under the standard ESI-MS conditions used for PAs (Figure 10). In our previous study, we used a method based on selected reaction monitoring by triple quadrupole to detect the PAs in non-oxidized and oxidized samples and noticed that the subunit composition changed due to the alkaline oxidation so that the method was not any longer capable of detecting the modified PAs [13]. The present high-resolution MS results support our previous observations by triple quadrupole that the modified PAs in the oxidized extracts are much more complex than the initial PAs in the non-oxidized extracts and that they are not detected under similar UPLC-MS/MS conditions [13].

The methods used for the oxidation of PAs in the literature are rather different from our method, and therefore, the results may not be directly comparable [14,24,25]. In previous studies, the oxidation has been performed in water acidified with trifluoroacetic acid at pH 3.5 in order to mimic the wine pH, and the actual oxidation has been obtained by stirring the sample in the presence of air for several days [14,24,25]. We used alkaline oxidation by carbonate buffer at pH 10 only for one hour.

Most of the plant extracts involved PAs with both PC and PD subunits, and only in two samples, the PAs could be considered almost PD pure. These two samples were the extracts of *Callisia gentlei* var. *elegans* and *Pellaea ovata*. The B-type PDs were detected in the *Callisia gentlei* var. *elegans* extract before the oxidation; see, for example, the small PD oligomers in Table S5, but after the oxidation, these PDs were not detected anymore by MS. We did neither detect any other signals that could correspond to the modified PDs nor to their possible degradation products. However, some of the modified PAs were still detected by UV at 280 nm as a lower and delayed hump (Figure S3). Interestingly, before the oxidation, A-type trimeric PDs were detected in the *Pellaea ovata* extract, and after the oxidation, these signals were still present, but their intensities were substantially lower by MS and, in addition, *m*/*z* values corresponding to the formation of additional A-type linkages in PD trimers were detected similarly to A-type PCs (Table S6). This may indicate that in addition to intermolecular reactions observed for B-type homo- and heterogenous PDs, the A-type PDs could also have similar intramolecular reactions that were detected for A-type PCs.

### 2.6. Galloylated PAs in Plant Extracts and Their Modifications Due to the Alkaline Oxidation

**Figure 11:**
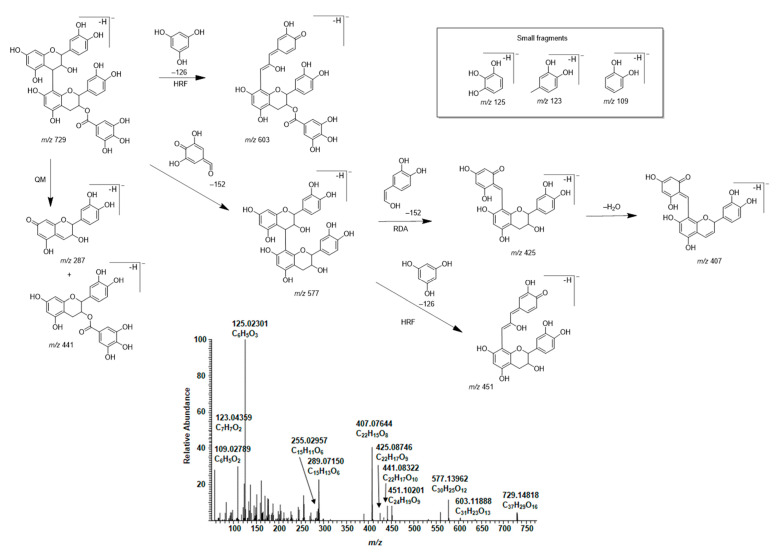
Characteristic fragmentation pathway and the MS/MS of a galloylated procyanidin dimer in the leaf extract of *Ruprechtia salicifolia*. The position of the galloyl group is only indicative and could be any free OH group in the terminal unit. The mechanisms are heterocyclic ring fission (HRF), retro-Diels‒Alder (RDA) fragmentation and quinone methide (QM) cleavage.

Some of the plants contained galloylated PAs. The presence of galloylated PCs, PDs and PC/PDs in plant extracts was confirmed by their characteristic fragmentation patterns. In general, the fragmentation of galloylated PAs in MS analysis occurred similarly via RDA, HRF and QM mechanisms as discussed above [31,32,33]. As an example, the characteristic fragmentation pathway and MS/MS data of a galloylated PC dimer in the leaf extract of *Ruprechtia salicifolia* are shown and discussed in detail (Figure 11). The position of the galloyl group is only indicative, and it could be attached to any other free hydroxyl group in the terminal unit. The ion at *m*/*z* 603 is the HRF product ion. The ions at *m*/*z* 287 and 441 corresponded to QM cleavage. In addition, we detected the cleavage of the galloyl group resulting in the ion corresponding for PC dimer at *m*/*z* 577 and the subsequent RDA fragmentation with an ion at *m*/*z* 425 and the further cleavage of water with an ion at *m*/*z* 407. The HRF of *m*/*z* 577 produced the fragment ion at *m*/*z* 451. In addition, we detected small fragment ions at *m*/*z* 109, 123 and 125, corresponding to the aromatic rings. The galloylated PAs could contain several galloyl groups in their structures (Table S7).

The different galloylated PAs behaved differently during the aerial oxidation under alkaline conditions. Galloylated PCs were rather stable and reacted similarly to nongalloylated PCs. For example, no notable changes were detected for the galloylated PCs having one galloyl group in their structures in the oxidized leaf extract of *Nepenthes maxima* (Table S7). However, it must be noted that the intensities of the ions for the galloylated PCs having two or more galloyl groups were lower in the oxidized extract of *Nepenthes maxima* and, for example, the galloylated PC pentamers with several galloyl groups were no longer detectable (Table S7). The integer *m*/*z* values for some galloylated PCs are similar to the *m*/*z* values for PC/PDs and, therefore, the ultrahigh-resolution MS is required. For example, *m*/*z* 881 corresponds to a trimeric PA consisting of two PC and one PD units (C_45_H_38_O_19_, mcalculated 882.20074) and to a galloylated PC dimer having two galloyl groups (C_44_H_34_O_20_, mcalculated, 882.16435). In some samples, such as in the oxidized leaf extract of *Coccoloba uvifera*, galloylated PCs were converted to A-type galloylated PCs due to oxidation, even though the galloylated PAs in the initial extract seemed to be similar to those in *Nepenthes maxima*. For example, the galloylated PC dimer having one galloyl group and exhibiting an ion *m*/*z* 729 (Table S7) was stable during the alkaline oxidation in the leaf extract of *Nepenthes maxima* but partly modified in the leaf extract of *Coccoloba uvifera* exhibiting ions both at *m*/*z* 727 and 729. The former ion corresponded for a galloylated A-type PC dimer (*m*/*z* 727.13148, C_37_H_27_O_16_) and showed characteristic MS/MS fragments supporting the A-type linkage: *m*/*z* 601 (HRF product of *m*/*z* 727), 575 (A-type PC dimer), 557 (cleavage of water from *m*/*z* 575), 449 (HRF of *m*/*z* 575, see Figure 4), 423 (RDA of *m*/*z* 575, see Figure 4), 285 (QM of *m*/*z* 575, see Figure 4), 169 (gallic acid), 125 (see Figure 11) and 109 (see Figure 11). One reason for these differences of similar PAs between plant extracts could be the other compounds present in the extracts and affecting the reactions of PAs.

Galloylated PC/PD mixtures and PDs were reactive and modified similarly to the nongalloylated PC/PD mixtures and PDs. After oxidation, these PAs were not detected by MS even though they were still visible at UV at 280 nm. We did neither detect any other signals that could correspond to these modified PAs nor to their possible degradation products. In one sample, namely in *Acacia karroo* leaves, the modified galloylated and nongalloylated PDs were not detected by UV at 280 nm either.

In our previous study [13], where PAs in non-oxidized and oxidized extracts were analyzed by selected reaction monitoring methods, we noticed that a clear galloyl hump was detected with the galloyl specific MS/MS method in the oxidized samples, but the shape of the hump had changed and shifted accordingly to the observed hump in the UV chromatogram. The original galloylated PAs had been modified in a way that the galloyl group could still be detected with the selected reaction monitoring method [13]. Previous studies on (-)-epigallocatechin gallate and (-)-epigallocatechin have shown that the trihydroxyphenyl B-ring is the principal site of action for oxidation and that there are no detectable products resulting from the oxidation of the galloyl moiety [42]. However, the reaction conditions used and oxidation products obtained were fairly different in comparison to our study as the oxidation was performed with peroxyl radicals generated by the thermolysis of the azo initiator 2,2′-azobis(2,4-dimethylvaleronitrile) [42].

In addition to galloylated PAs, two samples, namely *Cephalotaxus harringtonia* subsp. *drupacea* leaflets and *Laurus nobilis* leaves seemed to contain glycosylated PCs having one sugar unit attached to one PC structure. Some of these glycosylated B-type PCs were noticed to convert to glycosylated A-type PCs during the alkaline oxidation without the cleavage of the sugar unit. In addition, the formation of additional ether linkages to A-type PCs was detected.

## 3. Materials and Methods

The collection of plant materials, their processing and extraction, in addition, to oxidation at pH 10, were performed as previously reported [13]. The initial study contained 102 samples, of which we analyzed further altogether 55 samples (Table S8). For the aerial oxidation, 20 μL of each extract was oxidized with 180 μL of pH 10 buffer for 1 h at room temperature. Oxidation was stopped by adding 100 μL of 0.6% aqueous HCOOH. In addition, 280 μL of water was added to 20 μL of the initial non-oxidized prior to the analysis in order to get the end volume of 300 μL for both non-oxidized and oxidized samples. The ultrahigh-resolution mass spectrometric analysis was performed by a UPLC-DAD-ESI-QOrbitrap-MS/MS. The instrument consisted of an Acquity UPLC system (Waters Corp., Milford, MA, USA) coupled to a quadrupole–Orbitrap mass spectrometer (QExactiveTM, Thermo Fisher Scientific GmbH, Bremen, Germany). The column used was an Acquity UPLC BEH Phenyl column (2.1 × 100 mm, 1.7 μm, Waters Corp., Wexford, Ireland). The eluents were A = acetonitrile and B = 0.1% HCOOH. A flow rate of 0.5 mL min^−1^ was used, and the elution profile was as follows: 0‒0.5 min, 0.1% A in B (isocratic); 0.5‒5 min, 0.1‒30% A in B (linear gradient); 5‒6 min, 30%‒35% A in B (linear gradient); 6−6.1 min, 35‒90% A in B (linear gradient); 6.1−9.5 min, column wash and re-equilibration. The injection volume was 5 μL. The UV (λ = 190–500 nm) and MS data were recorded throughout the analysis. Negative ionization was used in heated ESI source with a spray voltage of −3.0 kV, sheath gas (N_2_) flow rate of 60, auxiliary gas (N_2_) flow rate of 20, sweep gas flow rate of 0, the capillary temperature of +380 °C and in-source collision-induced dissociation (CID) of 30 eV. For full scan MS, the mass range of orbitrap was *m*/*z* 150–2250, the resolution 35,000 and the automatic gain control 3 × 10^6^. For MS/MS analyses, namely dd-MS^2^ (TopN), the parameters were the following: TopN 3; the stepped normalized collision energies 20, 50 and 80 eV, the resolution 17,500 and the automatic gain control 1 × 10^5^. The calibration was performed by Pierce ESI Negative Ion Calibration Solution (Thermo Fisher Scientific Inc., Waltham, MA, USA). The data were processed with Thermo Xcalibur Qual Browser software (Version 3.0.63, Thermo Fisher Scientific Inc., Waltham, MA, USA). As the oxidized samples were obtained using sodium carbonate buffer (pH 10), sodium formed cluster ions with formic acid during the UHPLC-MS/MS analysis. Therefore, at the beginning of each total ion chromatogram of oxidized samples, a strong peak of sodium formate clusters was detected (Table S9). The pattern was easily detected and did not affect the analysis of PAs. MS/MS fragment ions of small PA oligomers are presented in Table S10.

## 4. Conclusions

In this study, we analyzed the PA composition of 55 plant extracts before and after alkaline oxidation by ultrahigh-resolution UHPLC-MS/MS (Table S8). The natural PA structures contained A- and B-type PCs, PDs and PC/PD mixtures in addition to galloylated ones. The PA compositions were complex, and the ultrahigh-resolution MS was needed to measure the exact masses and the corresponding molecular formulae of diverse PAs. B-type PCs in different plant extracts were rather stable and showed no or minor modification due to the alkaline oxidation. For some samples, we detected the intramolecular reactions of PCs producing A-type ether linkages. A-type PCs were also rather stable with no or minor modification, but, in some plants, the formation of additional ether linkages was detected. Plant extracts containing PD units in PAs (either pure PDs or PC/PD mixtures) were more reactive. After alkaline oxidation, these PAs or their oxidation products were no longer detected by MS even though a different type and/or delayed PA hump was still detected by UV at 280 nm. The intermolecular reactions between PAs probably modified these PAs so that they were not detected under the standard ESI-MS conditions. Previous studies have shown that these modified PAs cannot be studied either by degradation methods. Therefore, a new analytical method would be needed for their identification and characterization. Galloylated PAs were relatively stable under alkaline oxidation if they were PC-based, but additional ether linkages were formed supporting the conversion of galloylated B-type PAs to galloylated A-type PAs. PAs containing two or more galloyl groups were more reactive than those containing only one group. Galloylated PC/PD mixtures and PDs were more reactive and reacted similarly to nongalloylated ones. However, it must be noted that the intra- and intermolecular reactions were not exclusionary, and these reactions could occur simultaneously. In addition, the plant matrix and other compounds present affected these interactions.

## Data Availability

The data presented in this study are available on request from the corresponding author.

## Supplementary Materials

The following are available online, Figure S1: Extracted ion chromatograms at *m*/*z* 289.06–289.08, corresponding to flavan-3-ols (+)-catechin (rt = 2.93 min) and (−)-epicatechin (rt = 3.38 min) in (**A**) the non-oxidized extract and (B) oxidized extract of *Pavonia cauliflora* flowers, Figure S2: UV chromatograms at 280 nm of (**A**) the non-oxidized extract and (**B**) oxidized extract of *Podocarpus macrophyllus* leaves. The chromatographic humps corresponding for proanthocyanidins are highlighted with blue ovals, Figure S3: UV chromatograms at 280 nm of (**A**) the non-oxidized extract and (**B**) oxidized extract of *Pellaea ovata* pieces. The chromatographic humps corresponding for proanthocyanidins are highlighted with blue ovals, Table S1: The exact masses of the main ions of procyanidins in *Begonia bowerae* “Nigra” extract before (non-ox) and after the alkaline oxidation (ox). DP = degree of polymerization, Table S2: The exact masses of the main ions of A-type and B-type procyanidins in *Cyperus owanii* extract before (non-ox) and after the alkaline oxidation (ox). DP = degree of polymerization. The isotopic patterns of the [M‒H]^−^ and [M‒2H] ^2−^ ions of A-type trimer and hexamer, respectively, at *m*/*z* 863.18448 and the isotopic patterns of the [M‒H]^−^ and [M‒2H] ^2−^ ions of A-type tetramer and octamer, respectively, at *m*/*z* 1151.24678 are overlapping, Table S3: The exact masses of the main ions of A-type and B-type procyanidins in *Aglaonema commutatum* var. *maculatum* leaf extract before (non-ox) and after the alkaline oxidation (ox). DP = degree of polymerization, Table S4: The exact masses of the main ions of A-type and B-type proanthocyanidins containing both procyanidin (PC) and prodelphinidin (PD) units in the leaf extract of *Podocarpus macrophyllus* before (non-ox) and after the alkaline oxidation (ox). DP = degree of polymerization, Table S5: The exact masses of the main ions of B-type prodelphinidins (PDs) in the leaf extract of *Callisia gentlei var. elegans* before (non-ox) and after the alkaline oxidation (ox). None of the ions were detected after the alkaline oxidation (ox). DP = degree of polymerization, Table S6: The exact masses of the main ions of A-type prodelphinidins (PDs) in the extract of *Pellaea ovata* before (non-ox) and after the alkaline oxidation (ox), indicating the formation of additional A-type linkages during the oxidation. DP = degree of polymerization, Table S7: The exact masses of the main ions of galloylated procyanidins (PCs) in the leaf extract of *Nepenthes maxima* before (non-ox) and after the alkaline oxidation (ox). DP = degree of polymerization, G = galloyl group, * = not detected at all, Table S8: The plant species and parts studied before and after the alkaline oxidation by ultrahigh-performance liquid chromatography coupled to diode array detection and electrospray ionization quadrupole orbitrap tandem mass spectrometry. The UV (280 nm) and total ion chromatograms (TICs) are shown for non-oxidized and oxidized extracts with short insights into proanthocyanidin (PA) compositions and their changes* due to the alkaline oxidation. The first immense peak in the TICs of oxidized extracts corresponds to the sodium formate clusters formed during the analysis (Table S9). PA contents (mg/g), prodelphinidin (PD) shares, and mean degrees of polymerization (mDP) of PAs have been previously published in [13]**, Table S9: The sodium formate clusters formed during the ultrahigh-performance liquid chromatographic tandem mass spectrometric analysis. The pattern was easily detected and did not affect the analysis of proanthocyanidins, Table S10: MS/MS fragment ions of small proanthocyanidin oligomers detected by TopN method in the ultrahigh-performance liquid chromatographic tandem mass spectrometric analysis.

## Sample Availability

Samples of the compounds are not available from the authors.
