# Supplementary material for: Characterization of Natural and Alkaline-Oxidized Proanthocyanidins in Plant Extracts by Ultrahigh-Resolution UHPLC-MS/MS
Source: Molecules. 2021 Mar 26;26(7):1873. doi: 10.3390/molecules26071873 (PMC8037856; doi:10.3390/molecules26071873)
Supplement: Supplementary file 1 [file molecules-26-01873-s001.pdf]

Supplementary Material

Characterization of natural and alkaline-oxidized proanthocyanidins in plant extracts by ultrahigh-resolution UHPLC-MS/MS

Maarit Karonen *, Iqbal Bin Imran, Marica T. Engström and Juha-Pekka Salminen

Natural Chemistry Research Group, Department of Chemistry, University of Turku, FI-20014 Turku, Finland; iqbal.imran@utu.fi (I.B.I.) ; mtengs@utu.fi (M.T.E.); j-p.salminen@utu.fi (J.-P.S.)
* Correspondence: maarit.karonen@utu.fi; Tel.: +358-29-450-3179

Table of Contents

Figure S1 2
Figure S2 3
Figure S3 3
Table S1 4
Table S2 4
Table S3 5
Table S4 5
Table S5 6
Table S6 6
Table S7 7
Table S8 8
Table S9 36
Table S10 37

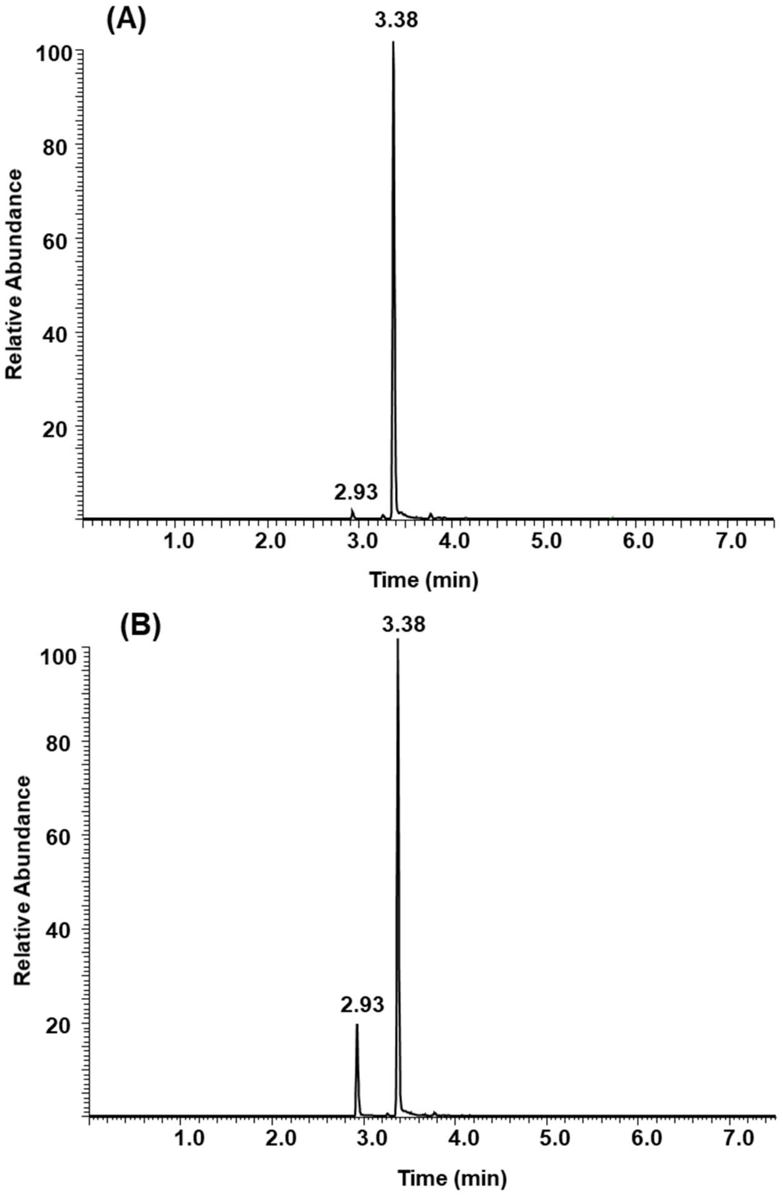

Figure S1. Extracted ion chromatograms at *m/z* 289.06-289.08 corresponding to flavan-3-ols (+)-catechin (rt = 2.93 min) and (−)-epicatechin (rt = 3.38 min) in (A) the non-oxidized extract and (B) oxidized extract of *Pavonia cauliflora* flowers.

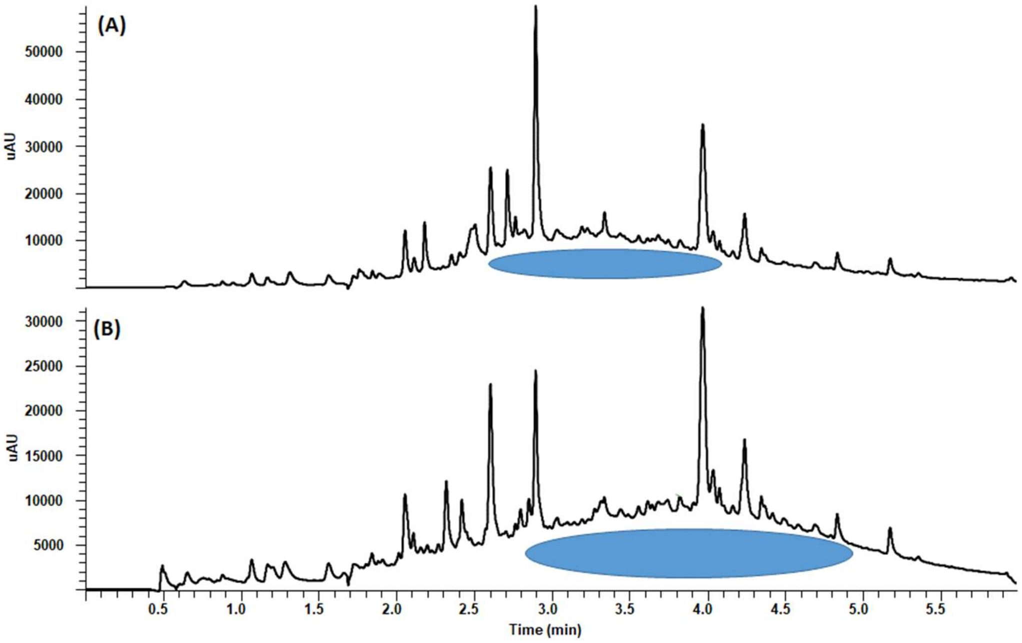

Figure S2. UV chromatograms at 280 nm of (A) the non-oxidized extract and (B) oxidized extract of *Podocarpus macrophyllus* leaves. The chromatographic humps corresponding for proanthocyanidins are highlighted with blue ovals.

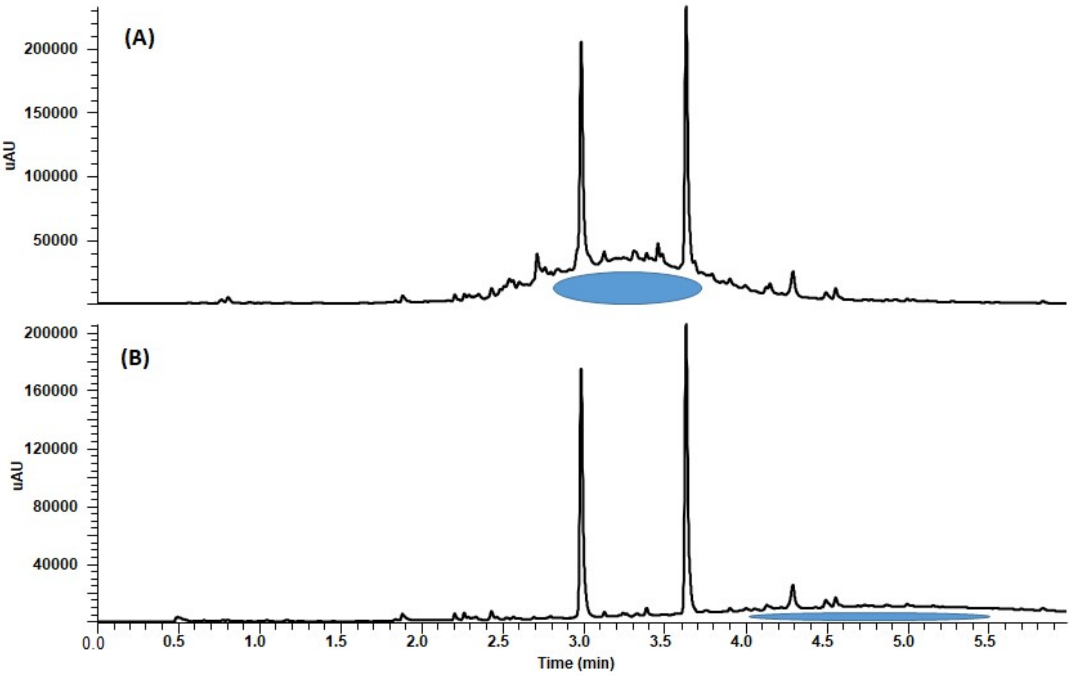

Figure S3. UV chromatograms at 280 nm of (A) the non-oxidized extract and (B) oxidized extract of *Pellaea ovata* pieces. The chromatographic humps corresponding for proanthocyanidins are highlighted with blue ovals.

Table S1. The exact masses of the main ions of procyanidins in *Begonia bowerae* "Nigra" extract before (non-ox) and after the alkaline oxidation (ox). DP = degree of polymerization.

			Observed m/z in non-ox		Observed m/z in ox	
DP	Molecular formula	Mcalculated	[M–H]⁻	[M–2H]²⁻	[M–H]⁻	[M–2H]²⁻
1	$C_{15}H_{14}O_6$	290.07904	289.07139		289.07154	
2	$C_{30}H_{26}O_{12}$	578.14243	577.13503		577.13542	
3	$C_{45}H_{38}O_{18}$	866.20582	865.19809		865.19948	
4	$C_{60}H_{50}O_{24}$	1154.26921	1153.26249		1153.26157	
5	$C_{75}H_{62}O_{30}$	1442.33260	1441.32551		1441.32652	
6	$C_{90}H_{74}O_{36}$	1730.39599	1729.38552		1729.38617	
7	$C_{105}H_{86}O_{42}$	2018.45938		1008.22377		1008.22351
8	$C_{120}H_{98}O_{48}$	2306.52277		1152.25393		1152.25272
9	$C_{135}H_{110}O_{54}$	2594.58616		1296.28772		1296.28773

Table S2. The exact masses of the main ions of A-type and B-type procyanidins in *Cyperus owanii* extract before (non-ox) and after the alkaline oxidation (ox). DP = degree of polymerization. The isotopic patterns of the [M–H]⁻ and [M–2H]²⁻ ions of A-type trimer and hexamer, respectively, at m/z 863.18448 and the isotopic patterns of the [M–H]⁻ and [M–2H]²⁻ ions of A-type tetramer and octamer, respectively, at m/z 1151.24678 are overlapping.

				Observed m/z in non-ox		Observed m/z in ox	
DP	Type	Molecular formula	Mcalculated	[M–H]⁻	[M–2H]²⁻	[M–H]⁻	[M–2H]²⁻
1		$C_{15}H_{14}O_6$	290.07904	289.07138		289.07162	
2	A	$C_{30}H_{24}O_{12}$	576.12678			575.12083	
2	B	$C_{30}H_{26}O_{12}$	578.14243	577.13513		577.13553	
3	A	$C_{45}H_{36}O_{18}$	864.19017			863.18448	
3	B	$C_{45}H_{38}O_{18}$	866.20582	865.19725		865.19855	
4	A	$C_{60}H_{48}O_{24}$	1152.25356			1151.24678	
4	B	$C_{60}H_{50}O_{24}$	1154.26921	1153.26124		1153.25940	
5	A	$C_{75}H_{60}O_{30}$	1440.31695			1439.31003	719.15276
5	B	$C_{75}H_{62}O_{30}$	1442.33260	1441.32451	720.15945	1441.32259	720.15945
6	A	$C_{90}H_{72}O_{36}$	1728.38034			1727.37496	863.18448
6	B	$C_{90}H_{74}O_{36}$	1730.39599	1729.38685	864.19053	1729.38685	864.18918
7	A	$C_{105}H_{84}O_{42}$	2016.44373			2015.42632	1007.21468
7	B	$C_{105}H_{86}O_{42}$	2018.45938	2017.44766	1008.22354	2017.44654	1008.22065
8	A	$C_{120}H_{96}O_{48}$	2304.50712				1151.24678
8	B	$C_{120}H_{98}O_{48}$	2306.52277		1152.25315		1152.25123
9	A	$C_{135}H_{108}O_{54}$	2592.57051				1295.27695
9	B	$C_{135}H_{110}O_{54}$	2594.58616		1296.28682		1296.28364

Table S3. The exact masses of the main ions of A-type and B-type procyanidins in *Aglaonema commutatum* var. *maculatum* leaf extract before (non-ox) and after the alkaline oxidation (ox). DP = degree of polymerization.

				Observed *m/z* in non-ox		Observed *m/z* in ox	
DP	Type	Molecular formula	Mcalculated	$[M-H]^-$	$[M-2H]^{2-}$	$[M-H]^-$	$[M-2H]^{2-}$
1		$C_{15}H_{14}O_6$	290.07904	289.07149		289.07164	
2	A	$C_{30}H_{24}O_{12}$	576.12678	575.11993		575.12012	
2	B	$C_{30}H_{26}O_{12}$	578.14243	577.13514			
3	A	$C_{45}H_{36}O_{18}$	864.19017	863.18318		863.18252	
4	A	$C_{60}H_{48}O_{24}$	1152.25356	1151.24529		1151.24507	
5	A	$C_{75}H_{60}O_{30}$	1440.31695	1439.30877		1439.30804	
6	A	$C_{90}H_{72}O_{36}$	1728.38034	1727.37308		1727.37069	
7	A	$C_{105}H_{84}O_{42}$	2016.44373	2015.43393	1007.21408	2015.43145	1007.21252
8	A	$C_{120}H_{96}O_{48}$	2304.50712		1151.24529		1151.24507
9	A	$C_{135}H_{108}O_{54}$	2592.57051		1295.77878		1295.77696

Table S4. The exact masses of the main ions of A-type and B-type proanthocyanidins containing both procyanidin (PC) and prodelphinidin (PD) units in the leaf extract of *Podocarpus macrophyllus* before (non-ox) and after the alkaline oxidation (ox). DP = degree of polymerization.

					Observed *m/z* in non-ox	Observed *m/z* in ox
DP	Monomeric units	Type	Molecular formula	Mcalculated	$[M-H]^-$	$[M-H]^-$
1	PC		$C_{15}H_{14}O_6$	290.07904	289.07149	289.07150
1	PD		$C_{15}H_{14}O_7$	306.07396	305.06648	
2	2PC	A	$C_{30}H_{24}O_{12}$	576.12678		575.12063
2	2PC	B	$C_{30}H_{26}O_{12}$	578.14243	577.13565	577.13633
2	PC+PD	B	$C_{30}H_{26}O_{13}$	594.13735	593.13069	
2	2PD	B	$C_{30}H_{26}O_{14}$	610.13226	609.12509	
3	3PC	A	$C_{45}H_{36}O_{18}$	864.19017		863.18654
3	3PC	B	$C_{45}H_{38}O_{18}$	866.20582	865.20014	
3	2PC+PD	B	$C_{45}H_{38}O_{19}$	882.20074	881.19370	
3	PC+2PD	B	$C_{45}H_{38}O_{20}$	898.19565	897.18826	
3	3PD	B	$C_{45}H_{38}O_{21}$	914.19057	913.18256	

Table S5. The exact masses of the main ions of B-type prodelphinidins (PDs) in the leaf extract of *Callisia gentlei var. elegans* before (non-ox) and after the alkaline oxidation (ox). None of the ions were detected after the alkaline oxidation (ox). DP = degree of polymerization.

				Observed *m/z* in non-ox	Observed *m/z* in ox
DP	Monomeric units	Molecular formula	Mcalculated	$[M–H]^-$	$[M–H]^-$
1	PD	$C_{15}H_{14}O_7$	306.07396	305.06658	
2	PC+PD	$C_{30}H_{26}O_{13}$	594.13735	593.13091	
2	2PD	$C_{30}H_{26}O_{14}$	610.13226	609.12535	
3	2PC+PD	$C_{45}H_{38}O_{19}$	882.20074	881.19394	
3	PC+2PD	$C_{45}H_{38}O_{20}$	898.19565	897.18877	
3	3PD	$C_{45}H_{38}O_{21}$	914.19057	913.18257	
4	2PC+2PD	$C_{60}H_{50}O_{26}$	1186.25904	1185.25200	
4	PC+3PD	$C_{60}H_{50}O_{27}$	1202.25396	1201.24662	
4	4PD	$C_{60}H_{50}O_{28}$	1218.24887	1217.23918	

Table S6. The exact masses of the main ions of A-type prodelphinidins (PDs) in the extract of *Pellaea ovata* before (non-ox) and after the alkaline oxidation (ox) indicating the formation of additional A-type linkages during the oxidation. DP = degree of polymerization.

				Observed *m/z* in non-ox	Observed *m/z* in ox
DP	Monomeric units	Molecular formula	Mcalculated	$[M–H]^-$	$[M–H]^-$
1	PD	$C_{15}H_{14}O_7$	306.07396	305.06664	
3	2PC+PD	$C_{45}H_{34}O_{19}$	878.16944		877.16025
3	2PC+PD	$C_{45}H_{36}O_{19}$	880.18509	879.17841	879.17299
3	PC+2PD	$C_{45}H_{34}O_{20}$	894.16435		893.15591
3	PC+2PD	$C_{45}H_{36}O_{20}$	896.18000	895.17317	895.16998
3	3PD	$C_{45}H_{34}O_{21}$	910.15927		909.15122
3	3PD	$C_{45}H_{36}O_{21}$	912.17492	911.16736	911.15793

Table S7. The exact masses of the main ions of galloylated procyanidins (PCs) in the leaf extract of *Nepenthes maxima* before (non-ox) and after the alkaline oxidation (ox). DP = degree of polymerization, G = galloyl group, * = not detected.

				Observed *m/z* in non-ox	Observed *m/z* in ox
DP	Monomeric units	Molecular formula	Mcalculated	$[M-H]^-$	$[M-H]^-$
1	PC	$C_{15}H_{14}O_6$	290.07904	289.07146	289.07152
1	PC+G	$C_{22}H_{18}O_{10}$	442.09000	441.08242	441.08234
2	2PC+G	$C_{37}H_{30}O_{16}$	730.15339	729.14605	729.14606
2	2PC+2G	$C_{44}H_{34}O_{20}$	882.16435	881.15723	881.15622
3	3PC+G	$C_{52}H_{42}O_{22}$	1018.21678	1017.20938	1017.20883
3	3PC+2G	$C_{59}H_{46}O_{26}$	1170.22774	1169.21955	1169.21937
3	3PC+3G	$C_{66}H_{50}O_{30}$	1322.23870	1321.22976	1321.23101
4	4PC+G	$C_{67}H_{54}O_{28}$	1306.28017	1305.26889	1305.26308
4	4PC+2G	$C_{74}H_{58}O_{32}$	1458.29113	1457.28071	1457.28115
4	4PC+3G	$C_{81}H_{62}O_{36}$	1610.30209	1609.29348	1609.28909
4	4PC+4G	$C_{88}H_{66}O_{40}$	1762.31305	1761.30325	1761.30252
5	5PC+G	$C_{82}H_{66}O_{34}$	1594.34356	1593.33218	1593.32330
5	5PC+2G	$C_{89}H_{70}O_{38}$	1746.35452	1745.34228	1745.34384
5	5PC+3G	$C_{96}H_{74}O_{42}$	1898.36548	1897.34980	*
5	5PC+4G	$C_{103}H_{78}O_{46}$	2050.37644	2049.35921	*
5	5PC+5G	$C_{110}H_{82}O_{50}$	2202.38740	2201.36825	*

Table S8. The plant species and parts studied before and after the alkaline oxidation by ultrahigh-performance liquid chromatography coupled to diode array detection and electrospray ionization quadrupole orbitrap tandem mass spectrometry. The UV (280 nm) and total ion chromatograms (TICs) are shown for non-oxidized and oxidized extracts with short insights into proanthocyanidin (PA) compositions and their changes* due to the alkaline oxidation. The first immense peak in the TICs of oxidized extracts corresponds to the sodium formate clusters formed during the analysis (Table S9). PA contents (mg/g), prodelphinidin (PD) shares and mean degrees of polymerization (mDP) of PAs have been previously published in [13]**.

No.	Plant Family and Species	Plant Part	PA Total (mg/g)**	PD %**	mDP**
	**Apocynaceae**				
1	*Mandevilla splendens*	leaves	28→26	0→1	10→11
	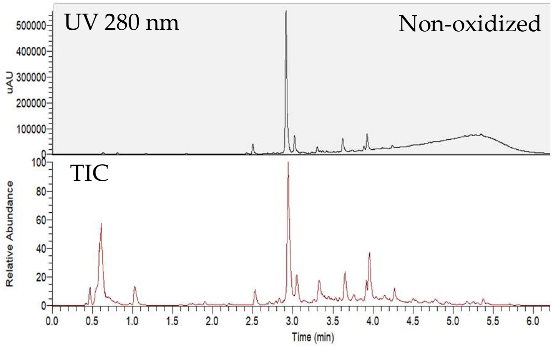	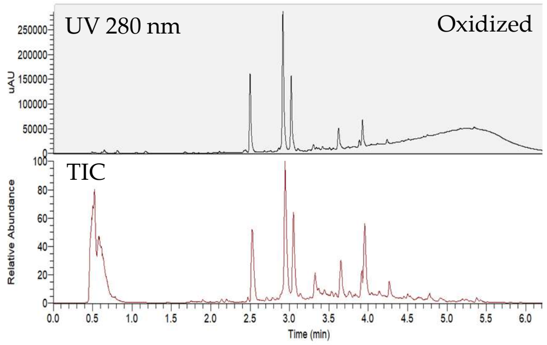			
	A-type PCs	no significant changes in PA composition			
	**Araceae**				
2	*Aglaonema commutatum var. maculatum*	leaves	19→10	0→0	4→3
	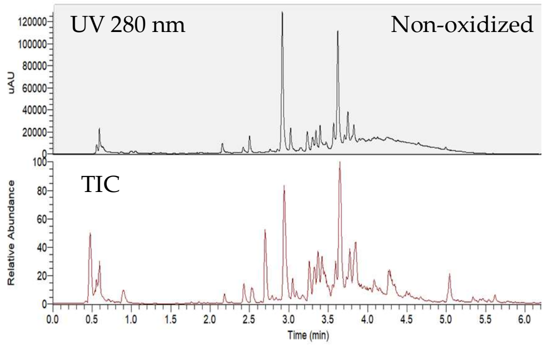	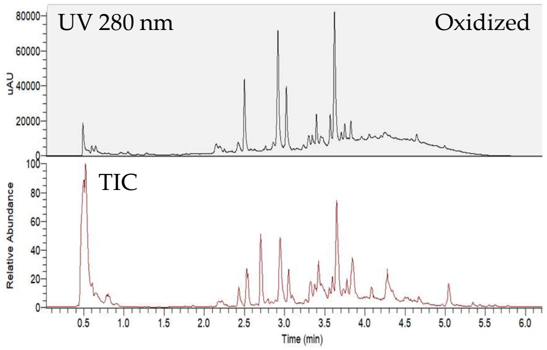			
	A-type PCs	no significant changes in PA composition			

No.	Plant Family and Species	Plant Part	PA Total (mg/g)**	PD %**	mDP**
3	*Aglaonema crispum*	leaves	13→1	0→0	4→2

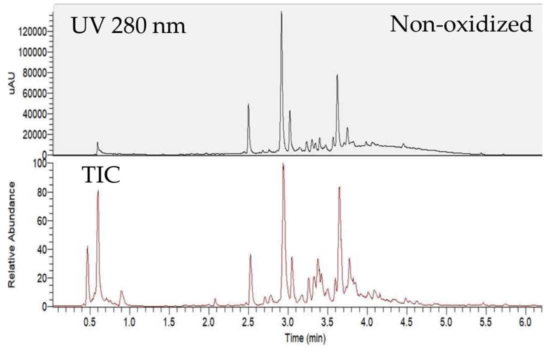

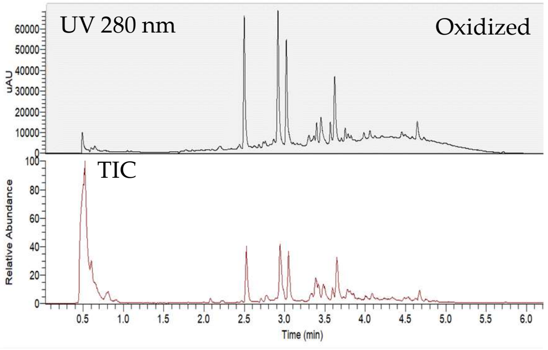

	A-type PCs, B-type PC dimer	formation of A-type linkages			
	**Araucariaceae**				
4	*Wollemia nobilis*	needles	19→12	1→0	9→9

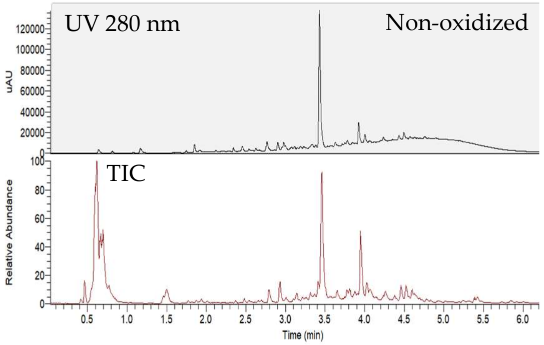

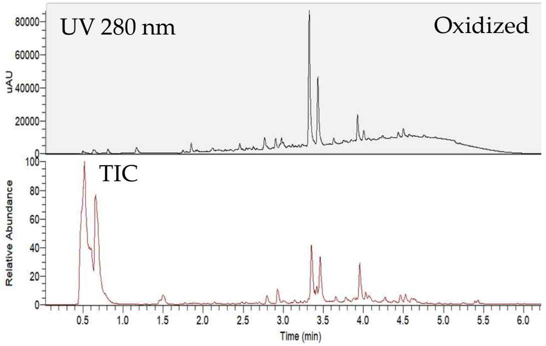

	B-type PCs	no significant changes in PA composition			

No.	Plant Family and Species	Plant Part	PA Total (mg/g)**	PD %**	mDP**
**5**	*Dianella intermedia*	leaves	7→1	50→0	10→5
	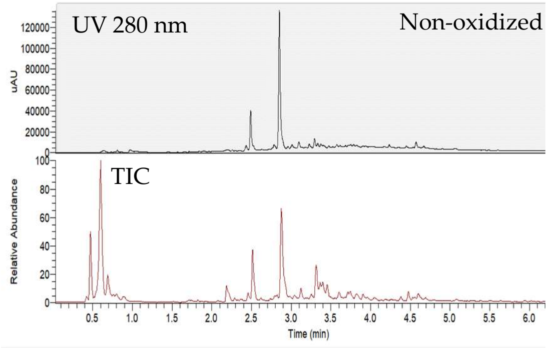	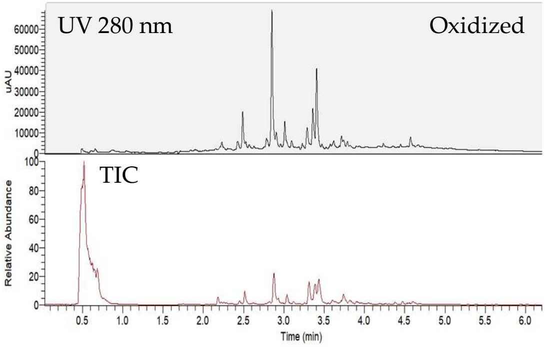			
	B-type PCs and PC/PDs	conversion from B- to A-type PCs, modification of PC/PDs			
	**Balsaminaceae**				
**6**	*Impatiens repens*	flowers	15→5	59→27	12→8
	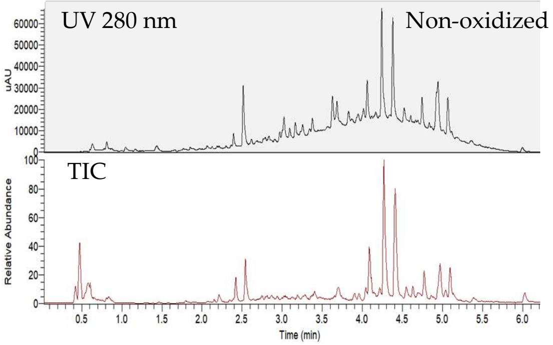	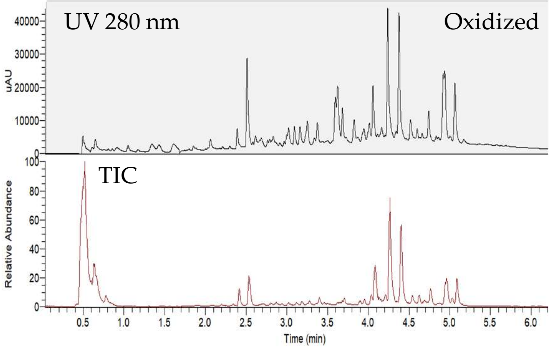			
	B-type PC/PDs	modification of PC/PDs			

No.	Plant Family and Species	Plant Part	PA Total (mg/g)**	PD %**	mDP**
7	*Begonia bowerae "Nigra"*	leaves	15→9	1→0	3→3

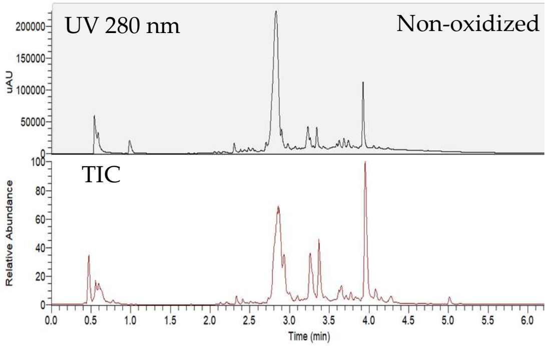

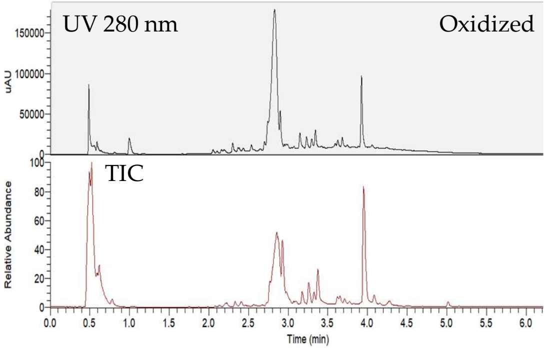

	B-type PCs	no significant changes in PA composition

No.	Plant Family and Species	Plant Part	PA Total (mg/g)**	PD %**	mDP**
	**Cephalotaxaceae**				
8	*Cephalotaxus harringtonia subsp. drupacea*	leaflets	55→22	8→8	3→3

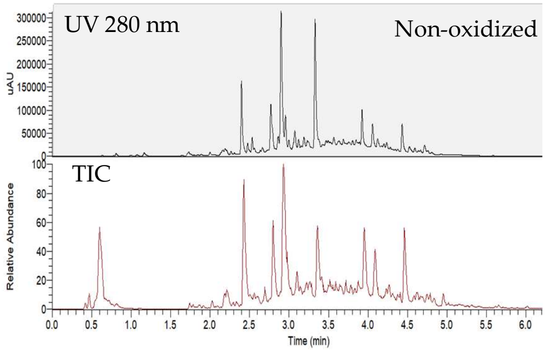

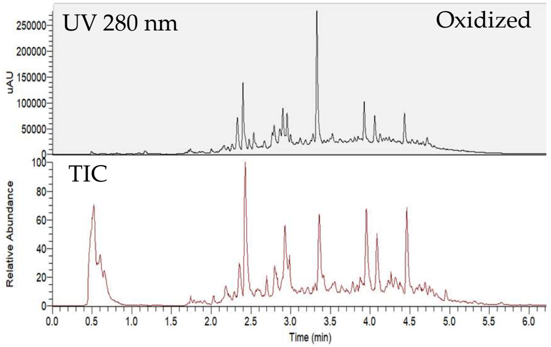

	B-type PCs and glycosylated PCs	conversion from B- to A-type PCs and galloylated PCs

No.	Plant Family and Species	Plant Part	PA Total (mg/g)**	PD %**	mDP**
	**Combretaceae**				
**9**	*Callisia gentlei var. elegans*	leaves	20→1	92→65	10→8

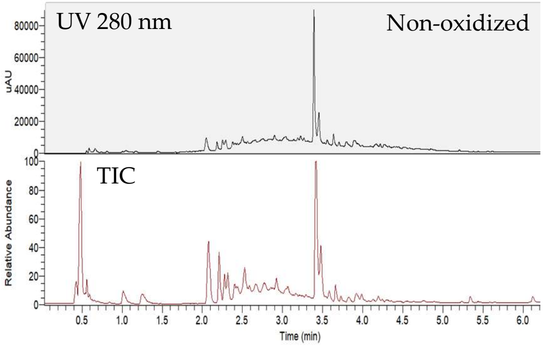

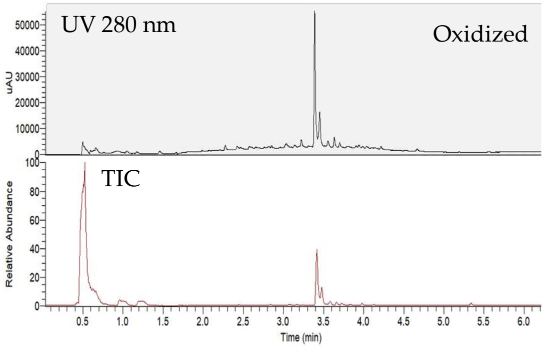

	B-type PDs	modifications of PDs			
**10**	*Combretum bracteosum*	leaves	11→1	0→0	4→3

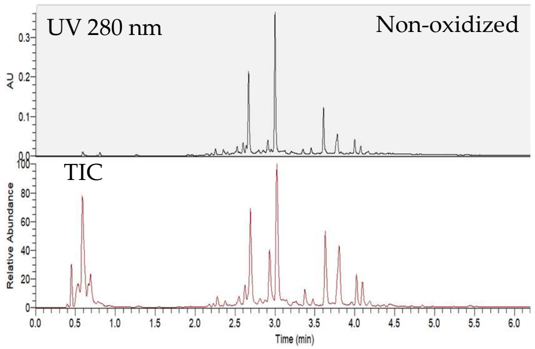

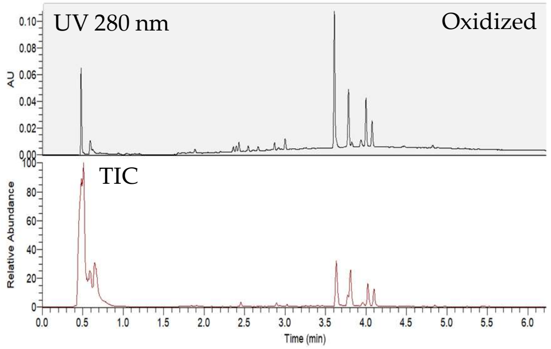

	B-type PCs	modifications of PCs

No.	Plant Family and Species	Plant Part	PA Total (mg/g)**	PD %**	mDP**
11	*Combretum indicum*	leaves	4→1	0→0	3→4
	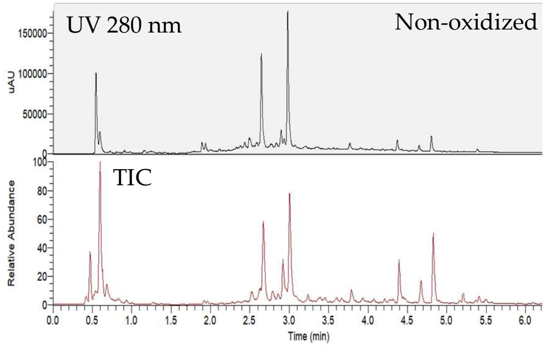	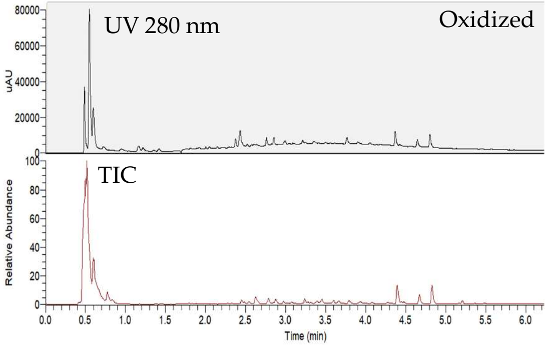			
	B-type PCs	modifications of PCs			
	**Cupressaceae**				
12	*Cunninghamia lanceolata*	leaves	51→34	1→1	4→4
	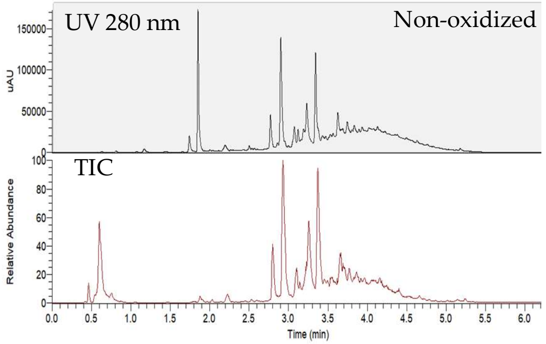	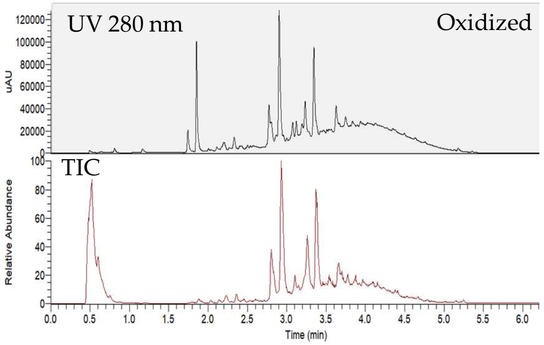			
	B-type PCs	no significant changes in PA composition			

No.	Plant Family and Species	Plant Part	PA Total (mg/g)**	PD %**	mDP**
13	*Sequoia sempervirens*	branches	25→1	82→0	11→2
	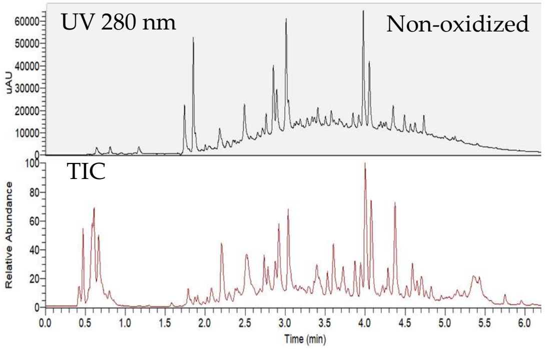	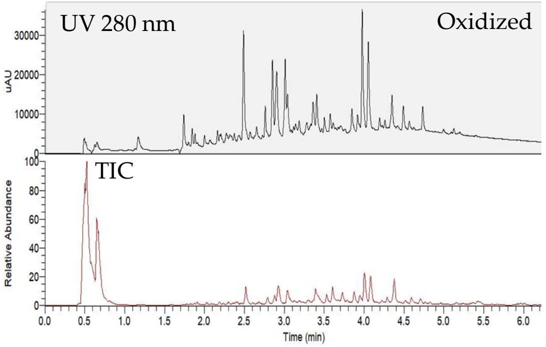			
	B-type PCs, PDs and PC/PDs	conversion from B- to A-type PCs, modification of PC/PDs and PDs			
	**Cyperaceae**				
14	*Cyperus owanii*	leaflets	19→9	6→0	6→6
	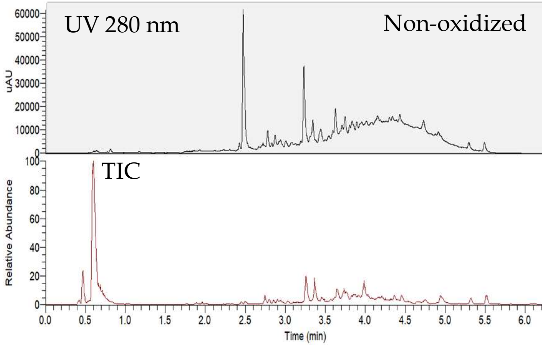	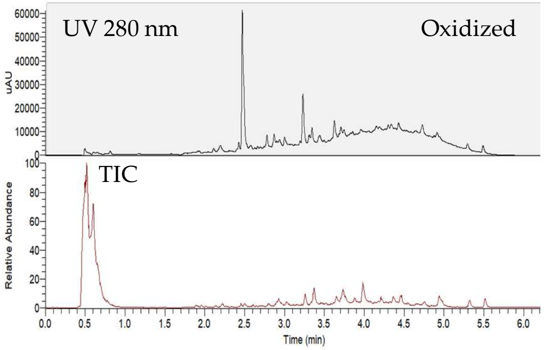			
	B-type PCs	conversion from B- to A-type PCs			

No.	Plant Family and Species	Plant Part	PA Total (mg/g)**	PD %**	mDP**
	**Dicksoniaceae**				
**15**	*Dicksonia squarrosa*	leaflets	20→8	2→2	3→2

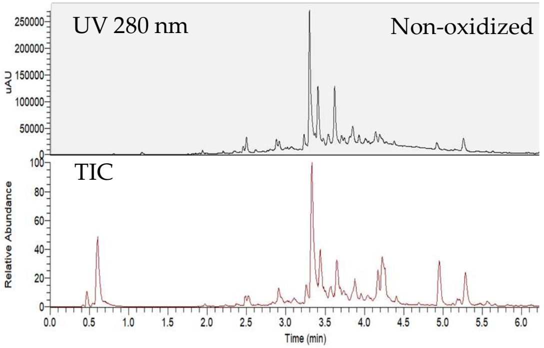

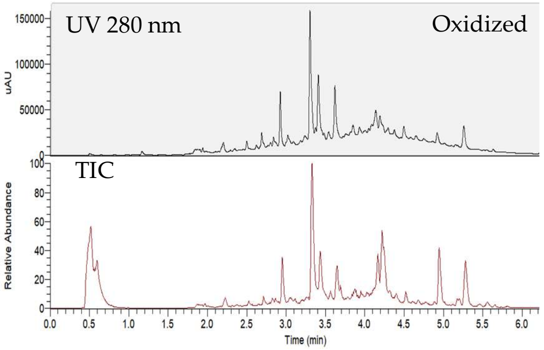

	A-type PCs, B-type PC dimer	formation of A-type linkages

No.	Plant Family and Species	Plant Part	PA Total (mg/g)**	PD %**	mDP**
	**Dryopteridaceae**				
**16**	*Polystichum proliferum*	leaves	44→25	1→2	13→13

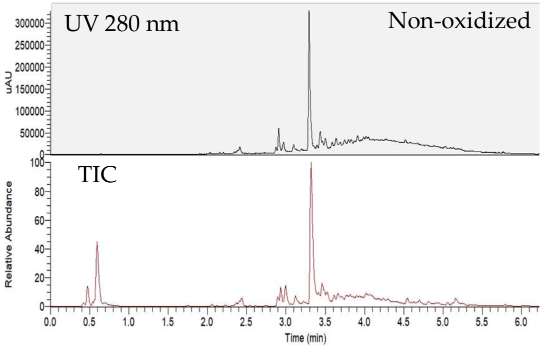

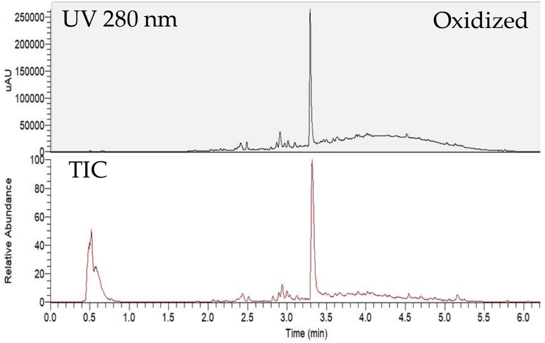

	A- and B-type PCs	minor conversion from B- to A-type PCs

No.	Plant Family and Species	Plant Part	PA Total (mg/g)**	PD %**	mDP**
	**Ericaceae**				
**17**	*Rhododendron hemitrichotum*	leaves	63→25	3→7	3→4

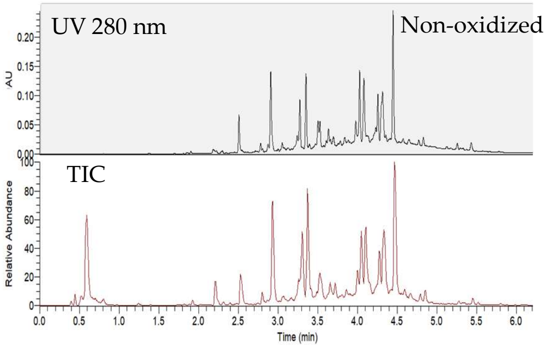

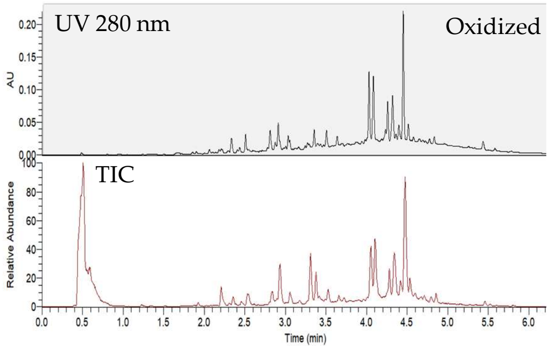

No.	Plant Family and Species	Plant Part	PA Total (mg/g)**	PD %**	mDP**
	A-type PCs, B-type PC dimer	additional A-type linkages detected, conversion from B- to A-type PC			
**18**	*Rhododendron hemitrichotum*	flowers	24→9	1→2	7→6

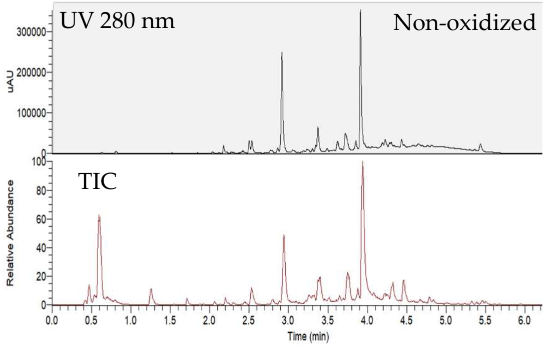

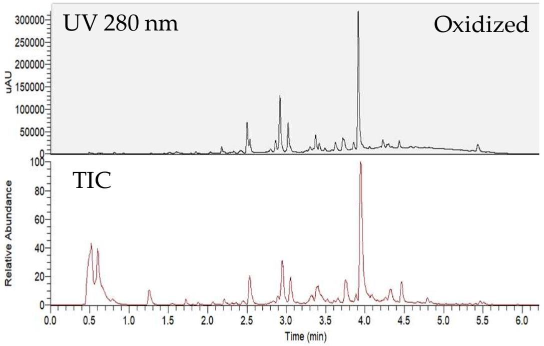

No.	Plant Family and Species	Plant Part	PA Total (mg/g)**	PD %**	mDP**
	A- and B-type PCs	additional A-type linkages detected, conversion from B- to A-type PCs			

No.	Plant Family and Species	Plant Part	PA Total (mg/g)**	PD %**	mDP**
	**Euphorbiaceae**				
**19**	*Euphorbia characias*	leaves	7→1	3→0	7→4

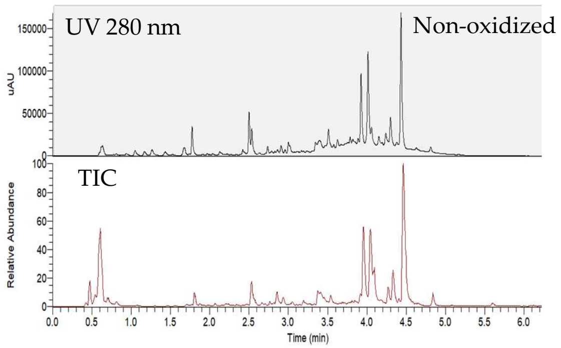

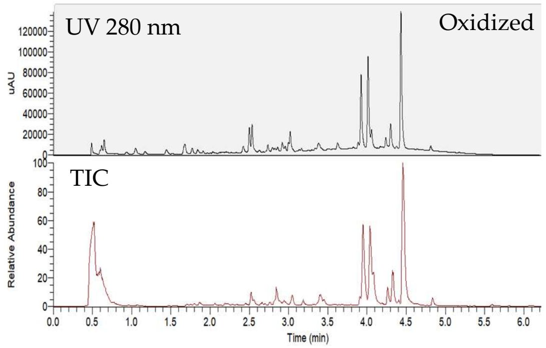

	B-type PCs	modifications of PCs, conversion from B- to A-type PCs			
	**Fabaceae**				
**20**	*Acacia karroo*	leaves	35→0	96→ND	10→ND

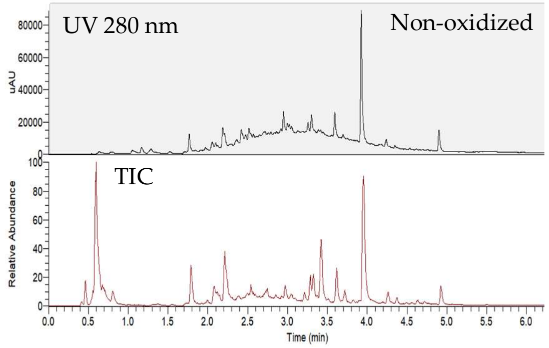

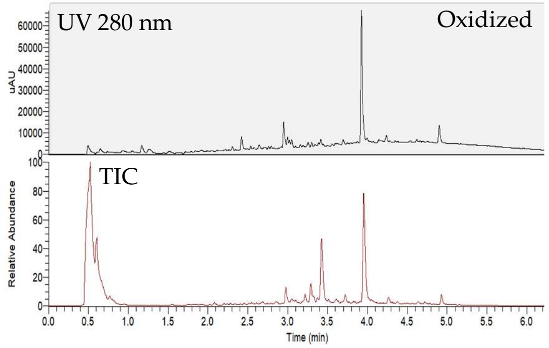

	B-type PDs and galloylated PDs	modifications of PDs and galloylated PDs			

No.	Plant Family and Species	Plant Part	PA Total (mg/g)**	PD %**	mDP**
21	*Acacia melanoxylon*	leaflets	47→8	56→4	8→4

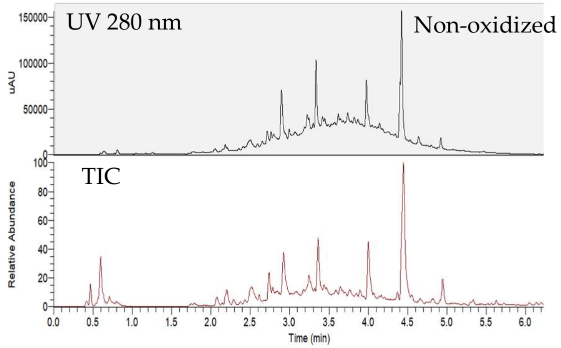

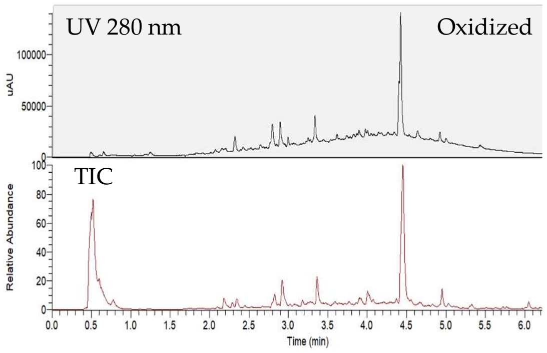

	B-type PCs, PDs, PC/PDs and galloylated PAs	conversion from B- to A-type PCs and galloylated PCs; modifications of PDs, PC/PDs and galloylated PDs and PC/PDs			
	**Lauraceae**				
22	*Apollonias barbujana*	leaves	30→20	0→1	3→3

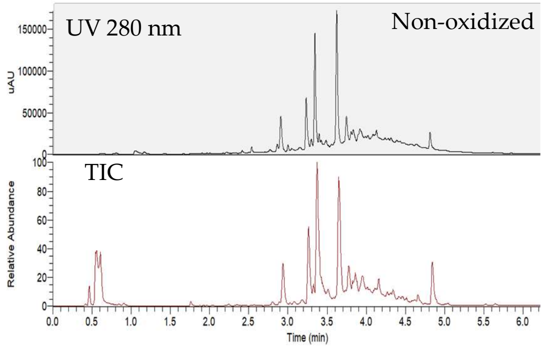

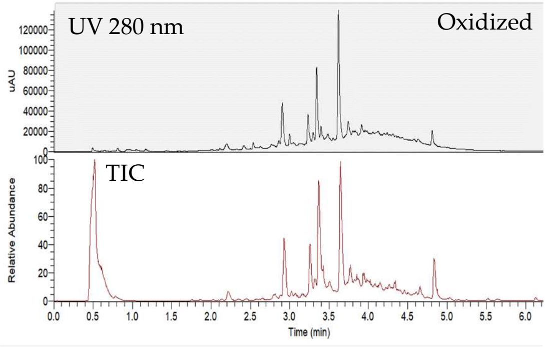

	A- and B-type PCs	no significant changes in PA composition			

No.	Plant Family and Species	Plant Part	PA Total (mg/g)**	PD %**	mDP**
23	*Laurus nobilis*	leaves	12→6	0→0	2→2

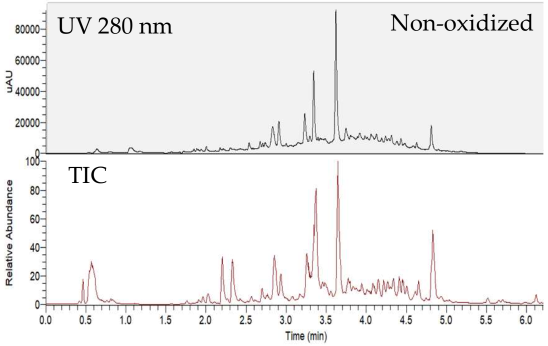

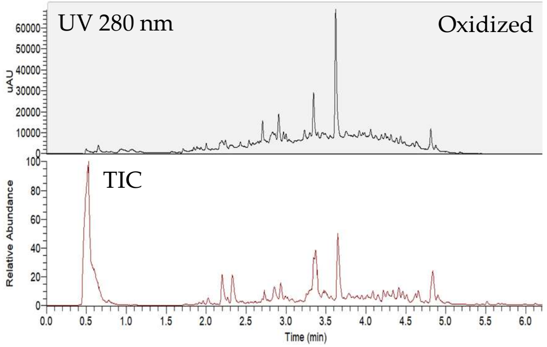

	A- and B-type PCs, glycosylated PCs	formation of A-type linkages			
	**Malvaceae**				
24	*Heritiera solomonensis*	leaves	76→50	0→1	7→7

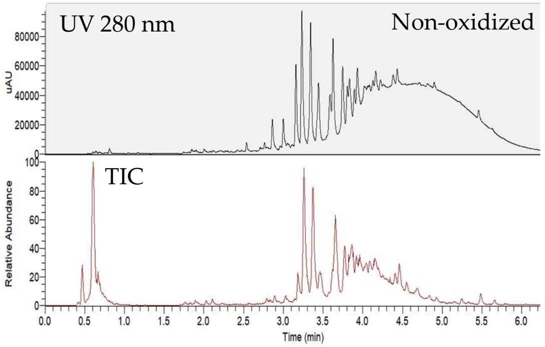

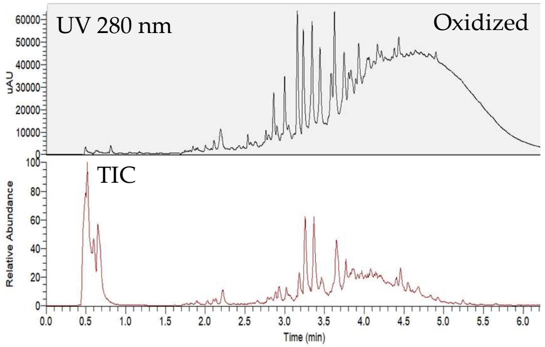

	B-type PCs	no significant changes in PA composition			

No.	Plant Family and Species	Plant Part	PA Total (mg/g)**	PD %**	mDP**
**25**	*Pavonia cauliflora*	flowers	25→12	1→1	6→6
	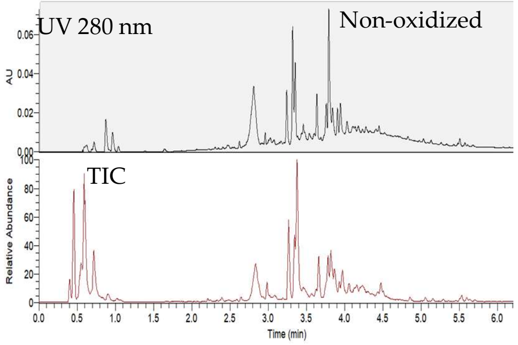	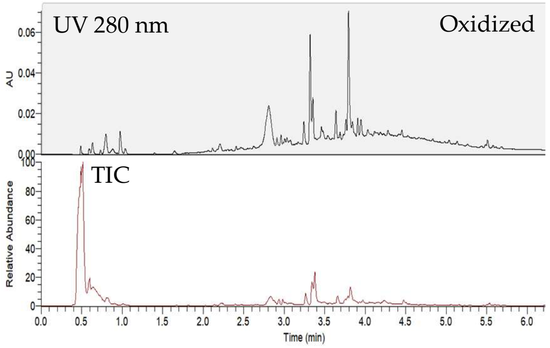			
	B-type PCs	conversion from B- to A-type PCs			
	**Marcgraviaceae**				
**26**	*Marcgravia umbellata*	leaves	29→13	0→2	3→3
	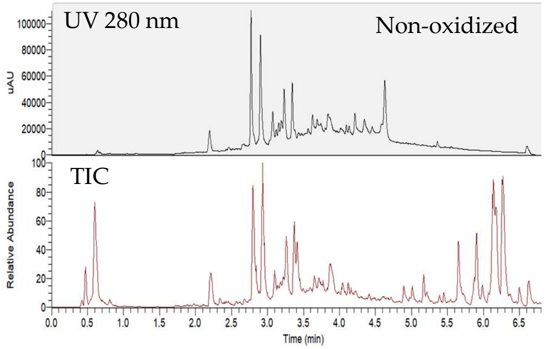	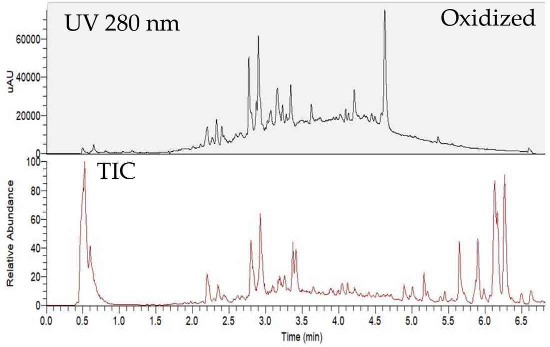			
	B-type PCs	conversion from B- to A-type PCs			

No.	Plant Family and Species	Plant Part	PA Total (mg/g)**	PD %**	mDP**
	**Nepenthaceae**				
**27**	*Nepenthes maxima*	leaves	22→6	2→5	4→3
	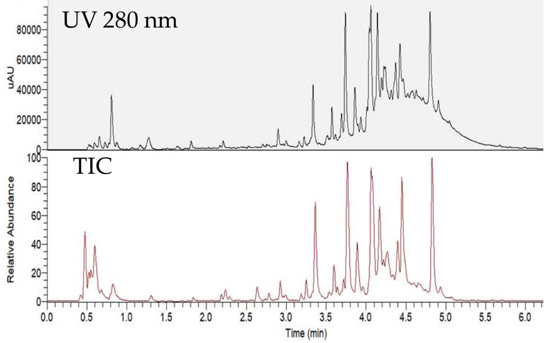	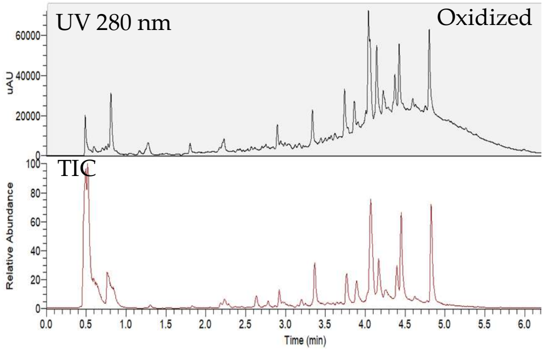			
	galloylated PCs	no significant changes in PA composition			
	**Oxalidaceae**				
**28**	*Biophytum sensitivum*	leaves	45→23	0→1	7→6
	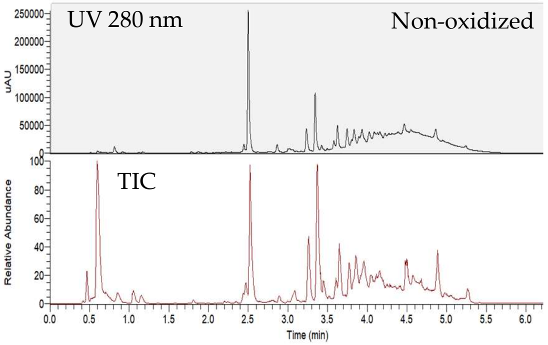	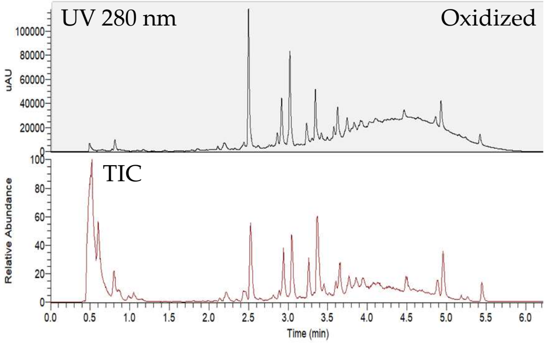			
	B-type PCs	conversion from B- to A-type PCs			

No.	Plant Family and Species	Plant Part	PA Total (mg/g)**	PD %**	mDP**
	**Podocarpaceae**				
**29**	*Podocarpus macrophyllus*	leaves	27→2	73→0	6→2
	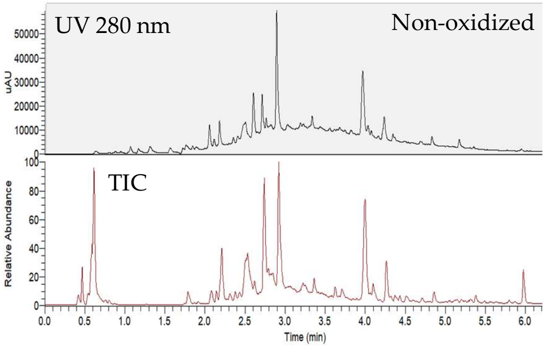	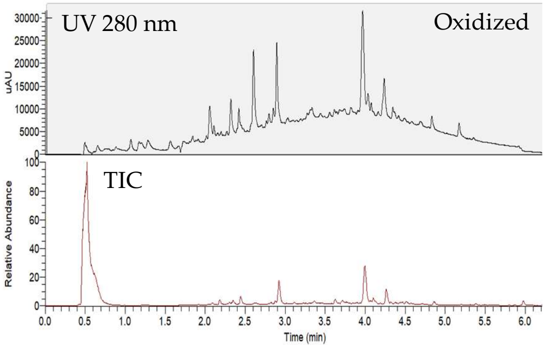			
	B-type PC/PDs and PDs	modifications of PC/PDs and PDs			
	**Polygonaceae**				
**30**	*Coccoloba uvifera*	leaves	24→9	14→3	6→4
	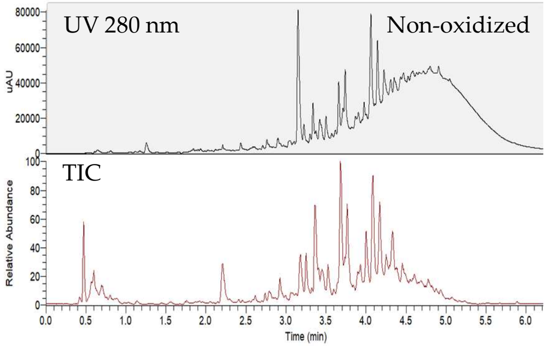	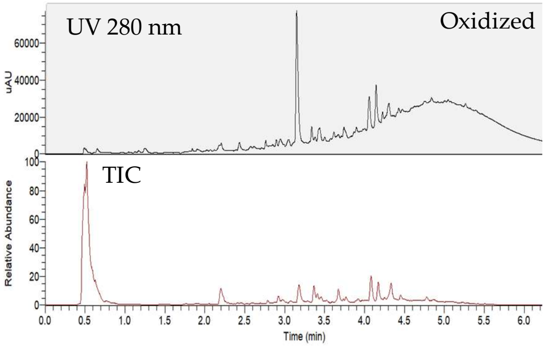			
	B-type PCs and galloylated PCs	conversion from B- to A-type PCs and galloylated PCs			

No.	Plant Family and Species	Plant Part	PA Total (mg/g)**	PD %**	mDP**
**31**	*Microgramma mauritiana*	leaflets	35→6	1→4	10→10

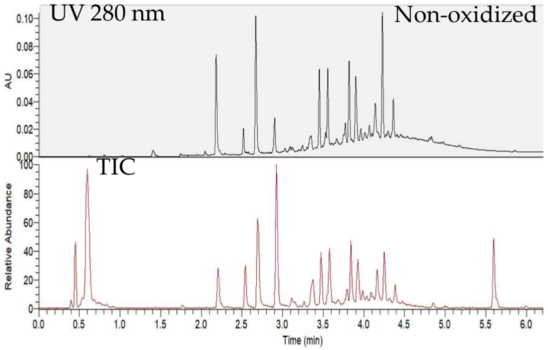

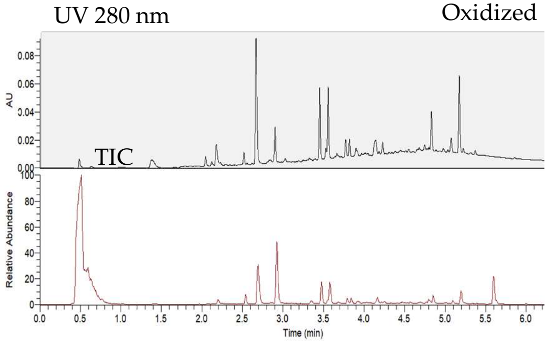

	B-type PCs	conversion from B- to A-type			

**32**	*Microgramma vacciniifolia*	leaves	11→2	0→0	4→3

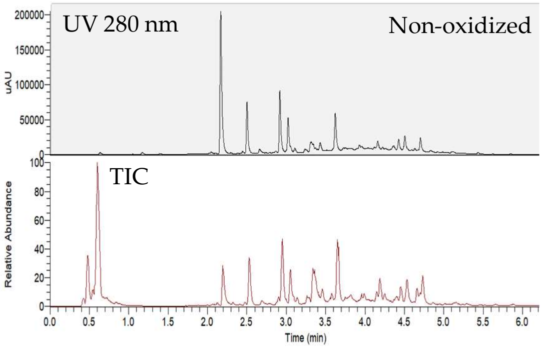

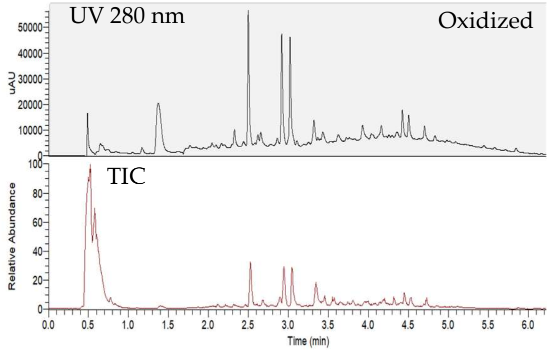

	A- and B-type PCs	modifications of A- and B-type PCs			

No.	Plant Family and Species	Plant Part	PA Total (mg/g)**	PD %**	mDP**
**33**	*Ruprechtia salicifolia*	leaves	24→4	3→5	7→4
	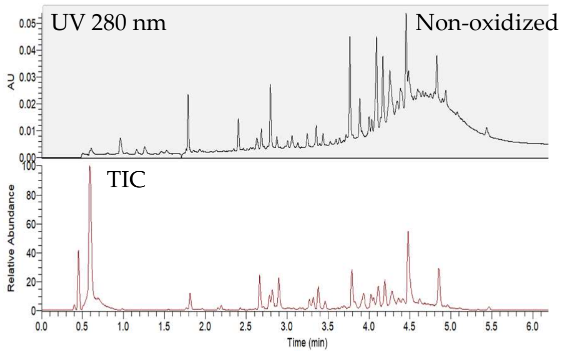	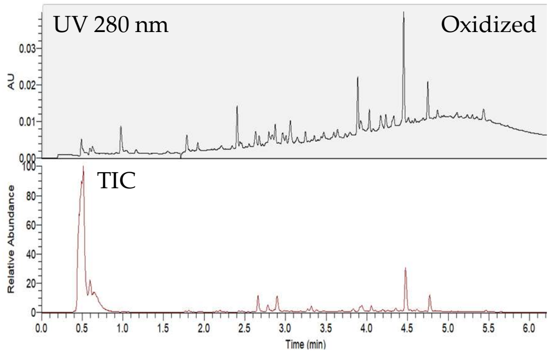			
	B-type galloylated PCs	conversion from B- to A-type galloylated PCs			
	**Portulacaceae**				
**34**	*Portulaca alata*	leaves	10→9	1→0	12→14
	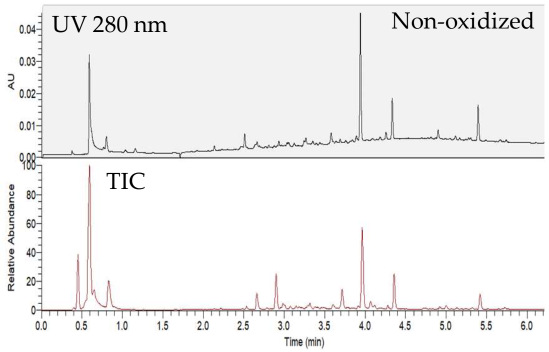	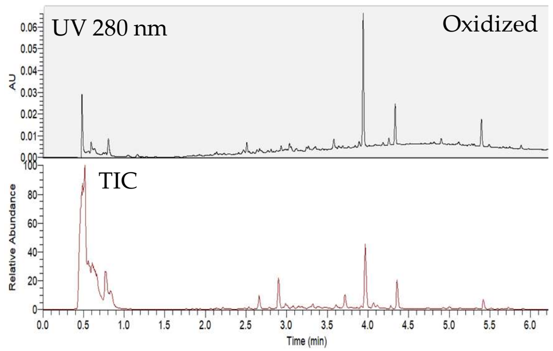			
	B-type PCs	no significant changes in PA composition			

No.	Plant Family and Species	Plant Part	PA Total (mg/g)**	PD %**	mDP**
	**Primulaceae**				
**35**	*Aegiceras corniculatum*	leaves	9→2	88→88	8→3

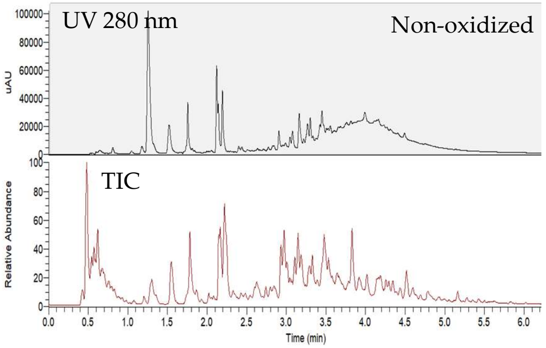

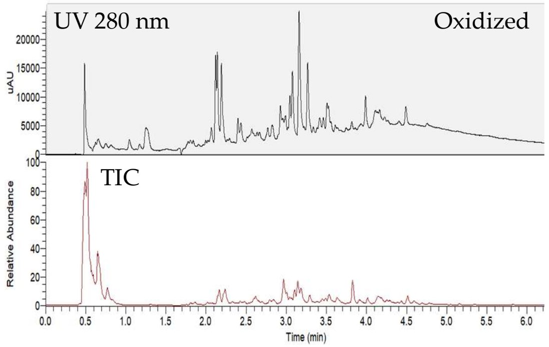

B-type galloylated PDs

modifications of galloylated PDs

No.	Plant Family and Species	Plant Part	PA Total (mg/g)**	PD %**	mDP**
**36**	*Ardisia crenata*	leaves	13→0	82→ND	12→ND

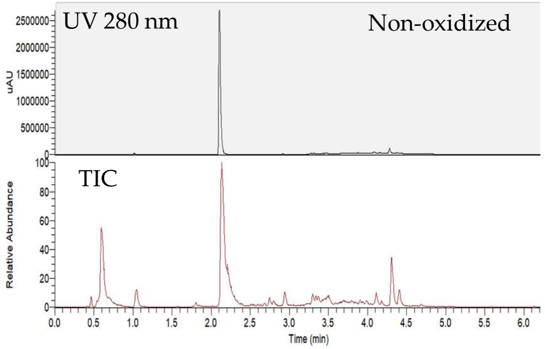

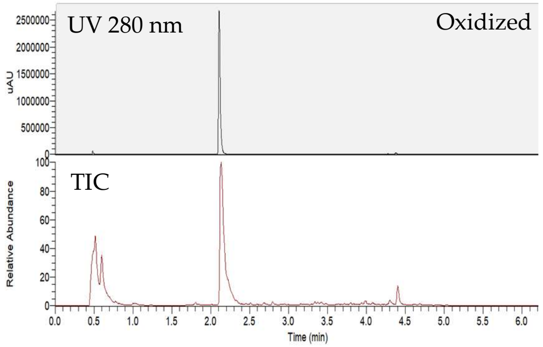

B-type galloylated PCs, PC/PDs and PDs

modifications of galloylated PAs

No.	Plant Family and Species	Plant Part	PA Total (mg/g)**	PD %**	mDP**
37	*Cyclamen africanum*	leaves	10→3	6→0	8→7
	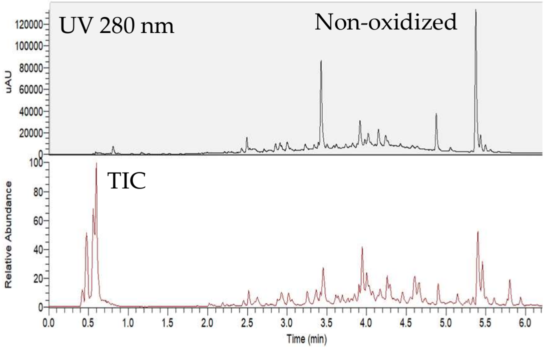	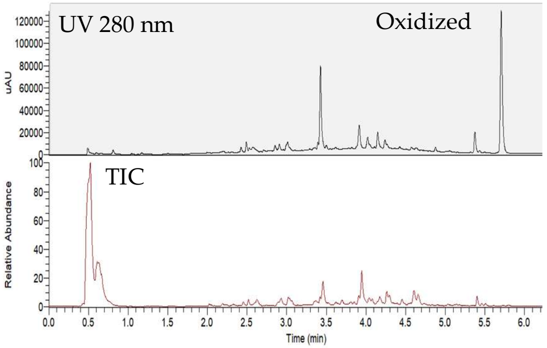			
	B-type PCs	conversion fom B- to A-type PCs			
	**Pteridaceae**				
38	*Pellaea ovata*	pieces	47→2	85→6	9→8
	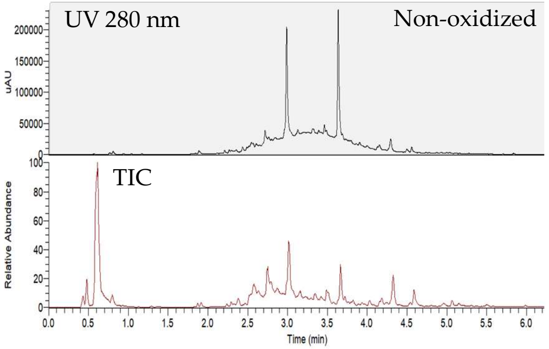	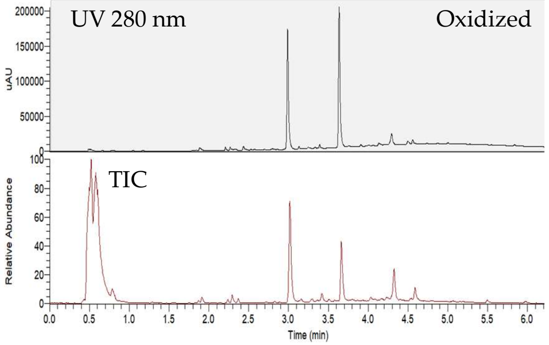			
	A-type PDs	modifications of PDs,<br>formation of additional A-type linkages			

No.	Plant Family and Species	Plant Part	PA Total (mg/g)**	PD %**	mDP**
	**Rhizophoraceae**				
**39**	*Rhizophora mangle*	leaves	35→9	4→3	6→6
	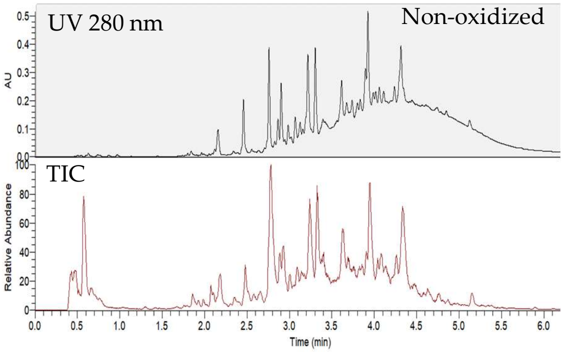	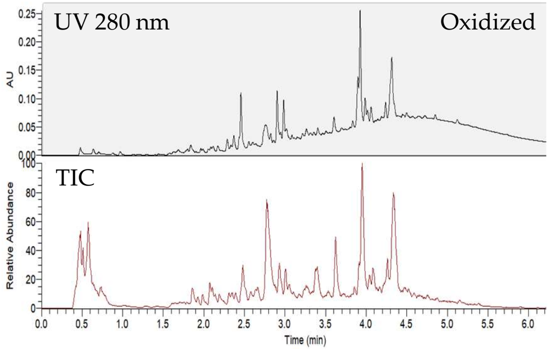			
	B-type PCs	conversion from B- to A-type PCs			
	**Rosaceae**				
**40**	*Osteomeles schweriniae*	leaves	37→17	1→1	8→8
	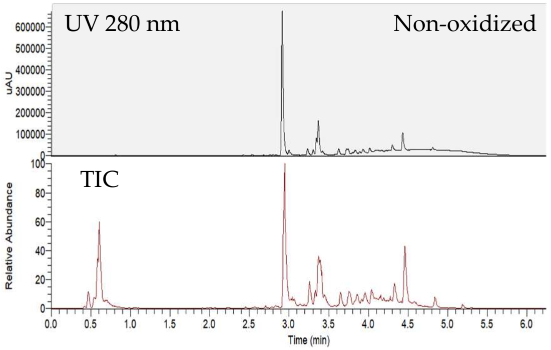	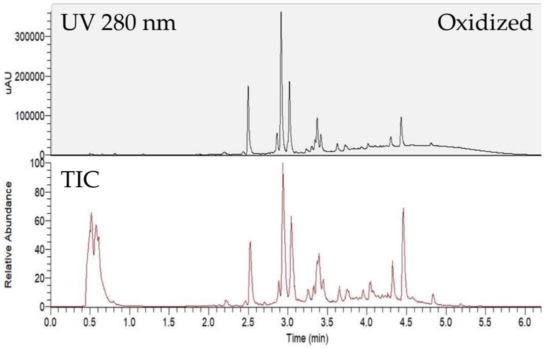			
	B-type PCs	conversion from B- to A-type PCs			

No.	Plant Family and Species	Plant Part	PA Total (mg/g)**	PD %**	mDP**
**41**	*Osteomeles schweriniae*	flowers	7→7	0→0	9→9

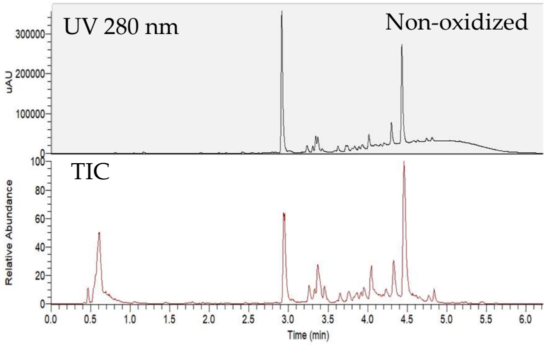

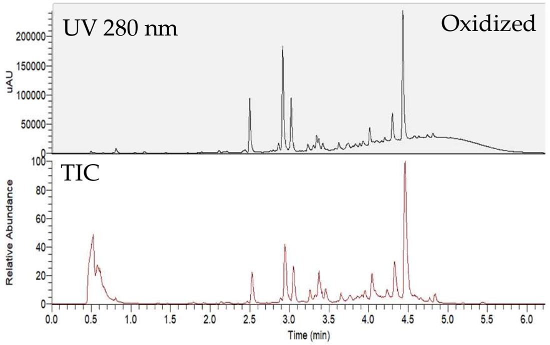

B-type PCs

no significant changes in PA composition

No.	Plant Family and Species	Plant Part	PA Total (mg/g)**	PD %**	mDP**
	**Rubiaceae**				
**42**	*Coffea arabica*	leaves	43→8	0→1	10→9

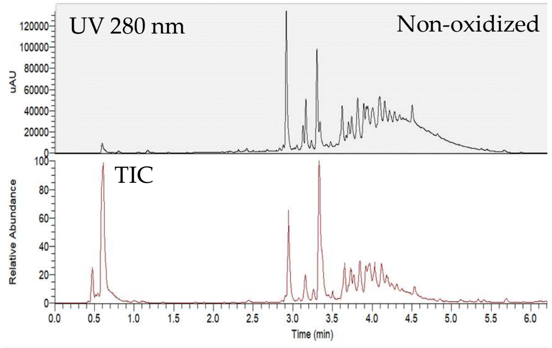

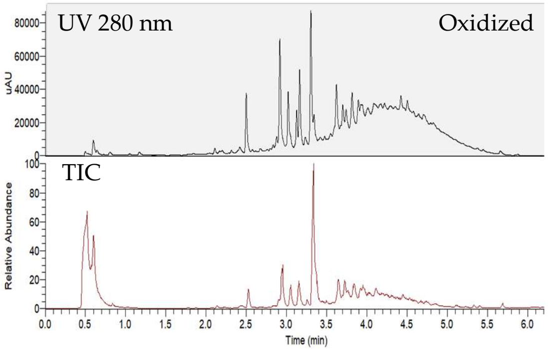

A- and B-type PCs

formation of A-type linkages

No.	Plant Family and Species	Plant Part	PA Total (mg/g)**	PD %**	mDP**
**43**	*Hoffmannia refulgens*	leaves	41→15	0→1	5→5

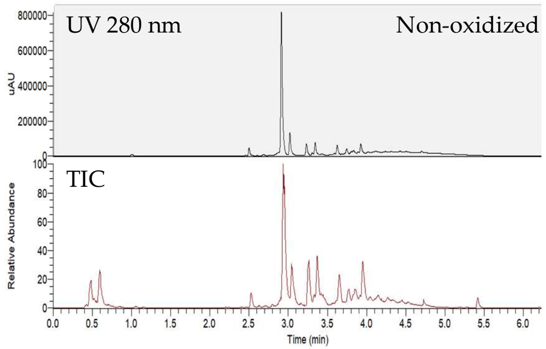

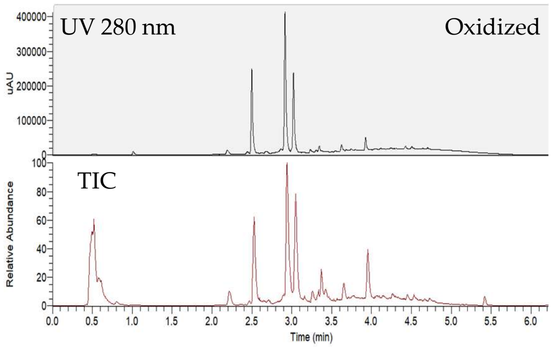

	B-type PCs	conversion from B- to A-type PCs			
**44**	*Ixora coccinea*	leaves	31→19	1→1	6→5

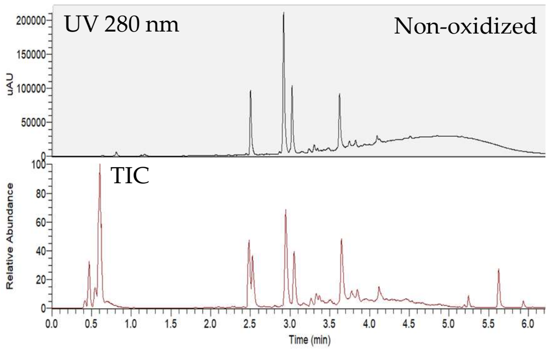

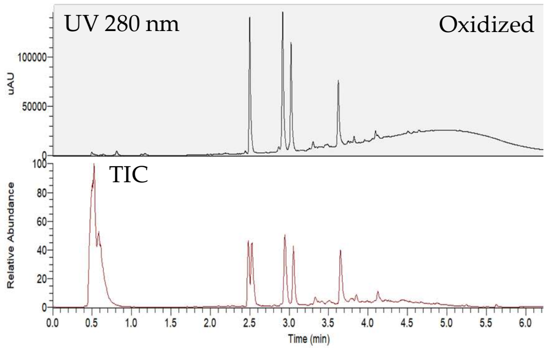

	A-type PCs	no significant changes in PA composition			

No.	Plant Family and Species	Plant Part	PA Total (mg/g)**	PD %**	mDP**
45	*Ixora coccinea*	flowers	16→10	0→0	7→6

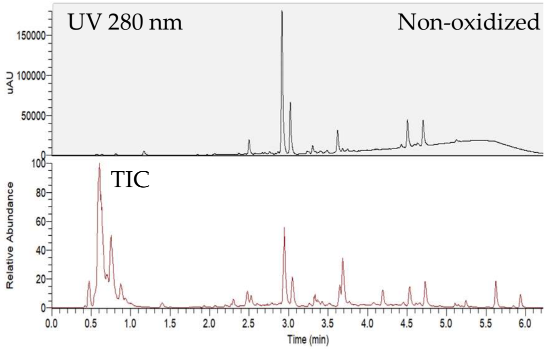

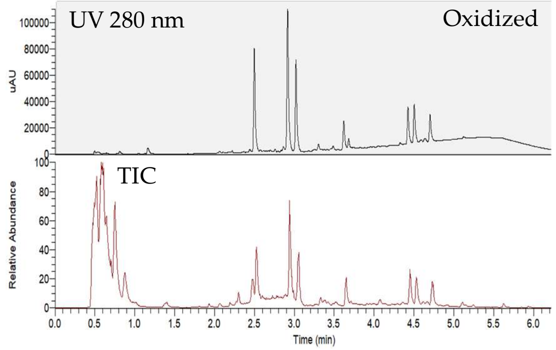

	A-type PCs	formation of additional A-type linkages			
	**Sarraceniaceae**				
46	*Sarracenia purpurea*	leaves	29→18	3→2	8→7

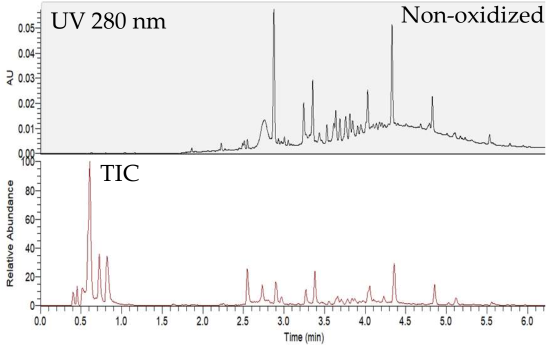

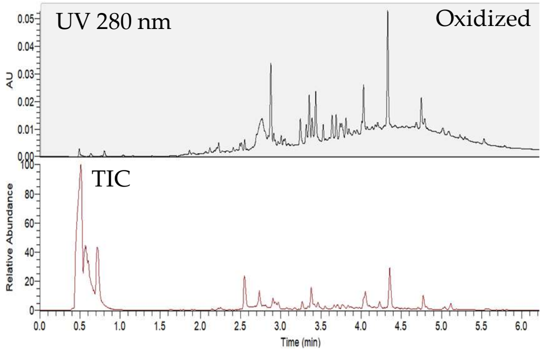

	B-type PCs	no significant changes in PA composition			

No.	Plant Family and Species	Plant Part	PA Total (mg/g)**	PD %**	mDP**
	**Strelitziaceae**				
**47**	*Strelitzia reginae*	leaves	9→6	0→0	5→5

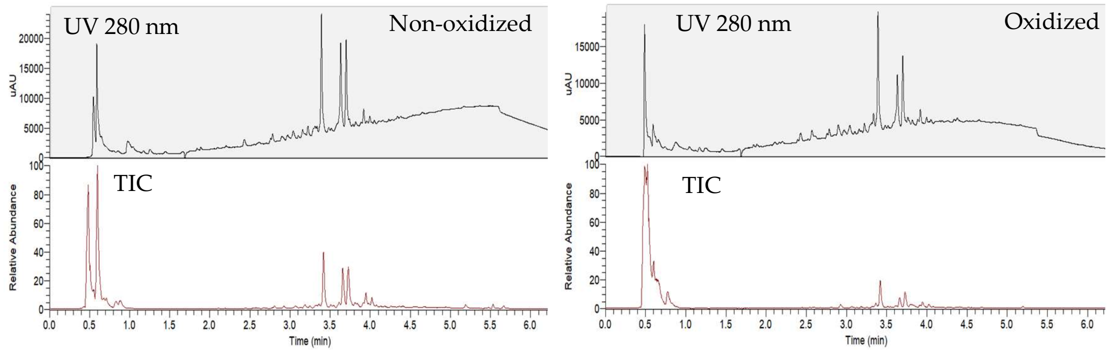

	A- and B-type PCs and unidentified PAs	no significant changes in PA composition

	**Tectariaceae**				
**48**	*Tectaria macrodonta*	leaflets	9→4	0→0	5→4

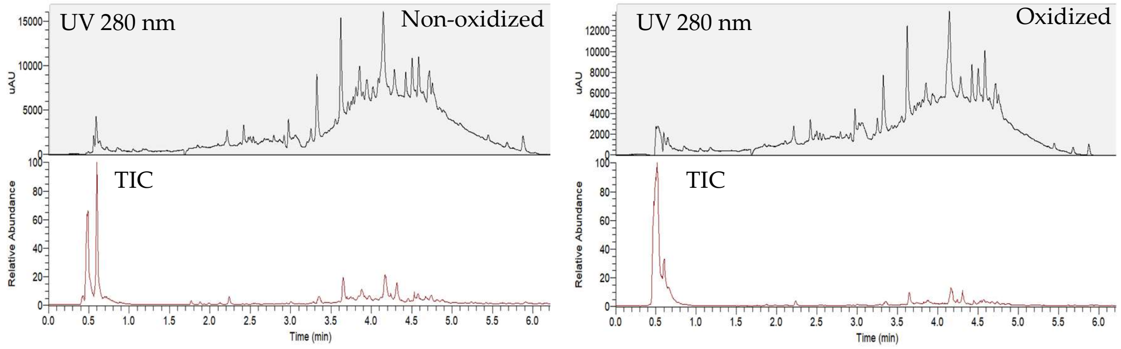

	A-type PCs	formation of additional A-type linkages

No.	Plant Family and Species	Plant Part	PA Total (mg/g)**	PD %**	mDP**
	**Theaceae**				
49	*Camellia japonica*	leaves	15→2	1→0	2→3
50	*Camellia japonica*	petals	18→10	1→2	3→3

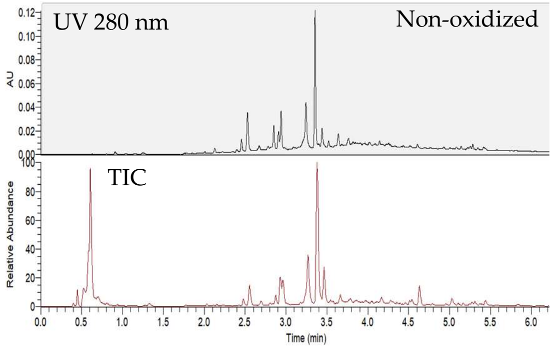

B-type PCs

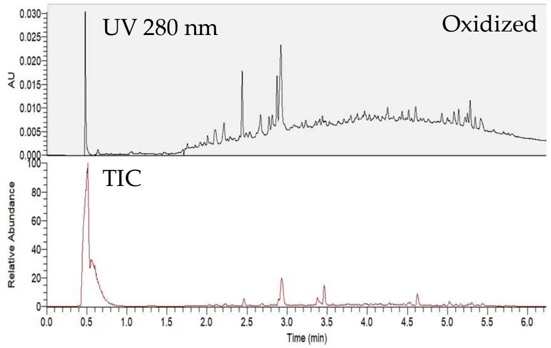

conversion from B- to A-type PCs

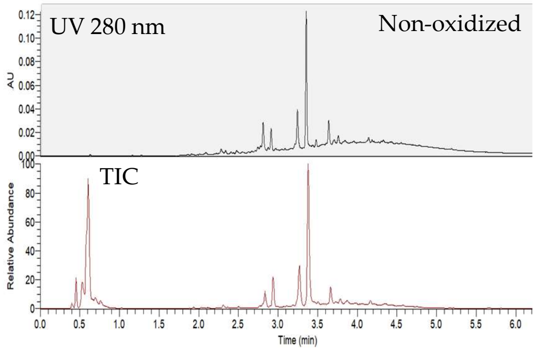

A- and B-type PCs

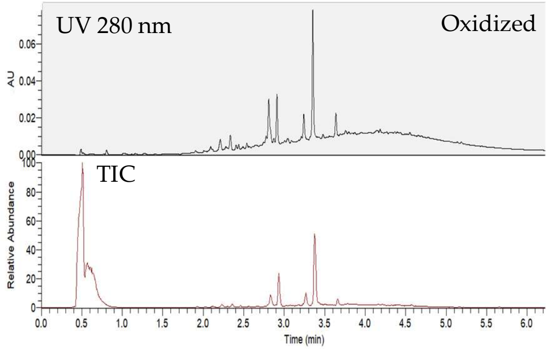

formation of A-type linkages

No.	Plant Family and Species	Plant Part	PA Total (mg/g)**	PD %**	mDP**
	**Vitaceae**				
**51**	*Rhoicissus sp.*	leaves	9→0	71→ND	10→ND
	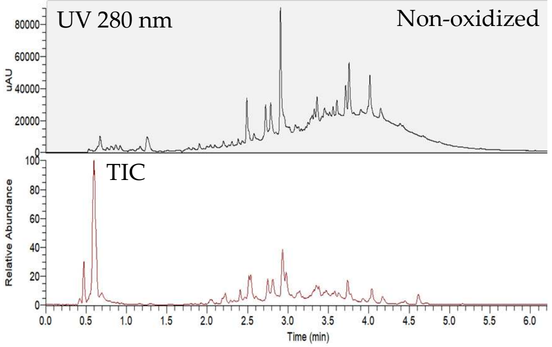	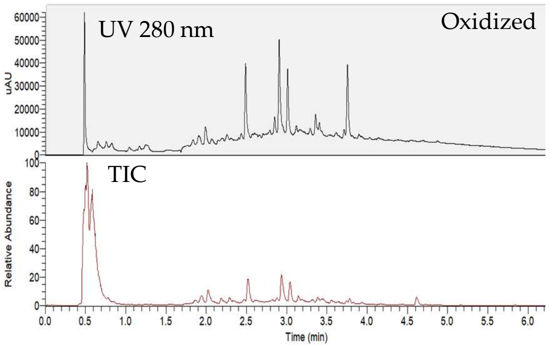			
	B-type galloylated PDs	modifications of galloylated PDs			
	**Zamiaceae**				
**52**	*Encephalartos ferox*	leaflets	21→13	0→0	9→9
	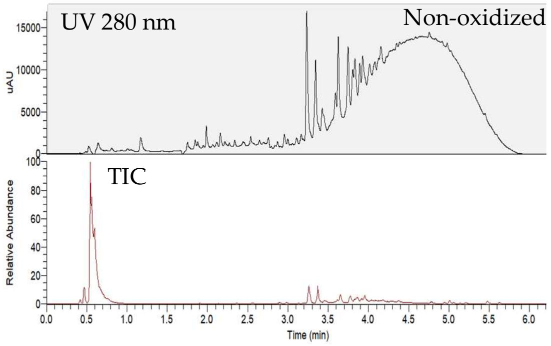	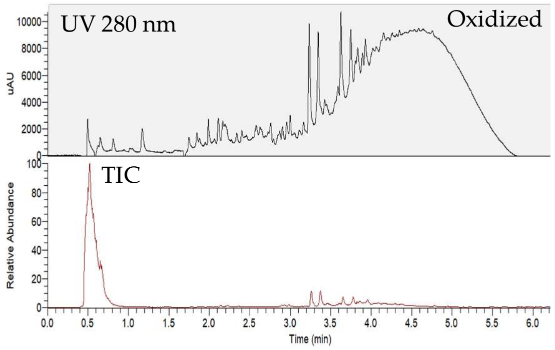			
	B-type PCs	conversion from B- to A-type PCs			

No.	Plant Family and Species	Plant Part	PA Total (mg/g)**	PD %**	mDP**
53	*Macrozamia communis*	leaflets	41→30	1→1	9→9
	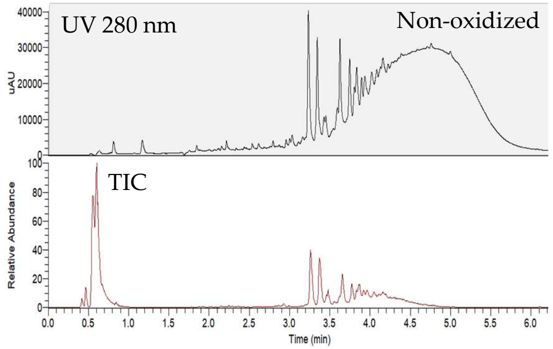	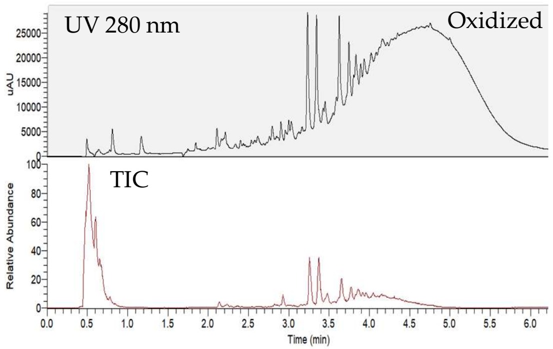			
	B-type PCs	no significant changes in PA composition			
	**Zingiberaceae**				
54	*Alpinia purpurata*	leaves	34→17	0→1	7→7
	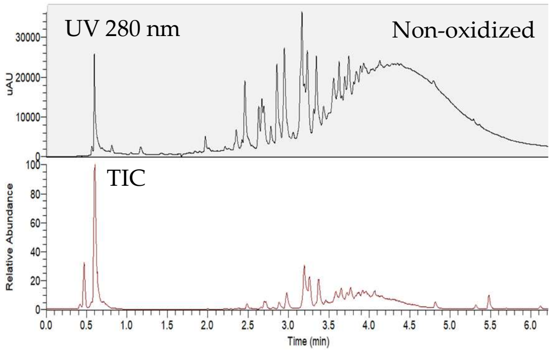	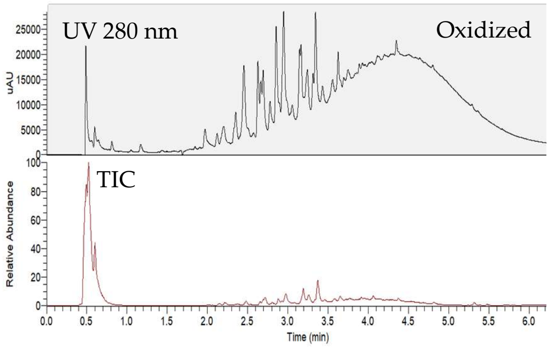			
	B-type PCs	conversion from B- to A-type PCs			

No.	Plant Family and Species	Plant Part	PA Total (mg/g)**	PD %**	mDP**
**55**	*Elettaria cardamomun*	leaflets	21→8	0→2	5→5
	B-type PCs	conversion from B- to A-type PCs			

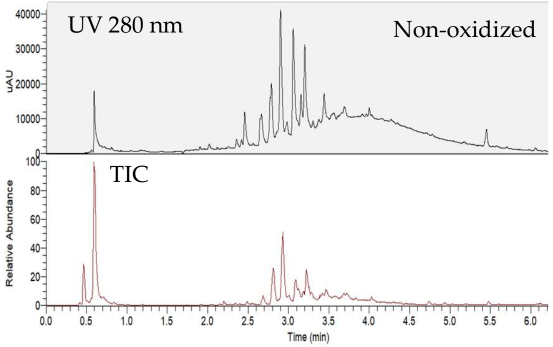

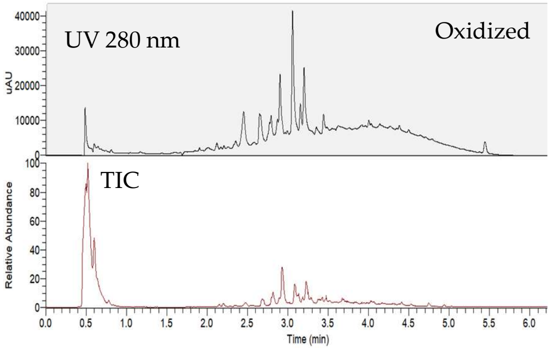

*) The main changes are described. There can be other modification reactions present too. In addition, it must be noted that modification does not mean that all PAs would behave like this. For example, "conversion from B-type to A-type PCs" means that this kind of modification is detected but the degree of conversion varies and in most cases, only a part of B-type PCs are converted to A-type PCs.

**) The quantitative and qualitative measures (PA total content (mg/g), PD % and mDP) before and after oxidation have been previously published in Imran et al. (2021): https://pubs.acs.org/doi/full/10.1021/acsomega.0c05515 and reused by the permission of ACS. Further permission related to the material excerpted should be directed to the ACS. The PA contents were determined by selected reaction monitoring methods for PCs and PDs. The method cannot detect the monomeric units that have the ether linkage characteristic for A-type PAs. ND = not detected.

Table S9. The sodium formate clusters formed during the ultrahigh-performance liquid chromatographic tandem mass spectrometric analysis. The pattern was easily detected and did not affect the analysis of proanthocyanidins.

m/z	Cluster ion
180.97	$HCOO(NaCOOH)_2$
248.96	$HCOO(NaCOOH)_3$
316.95	$HCOO(NaCOOH)_4$
384.93	$HCOO(NaCOOH)_5$
452.92	$HCOO(NaCOOH)_6$
520.91	$HCOO(NaCOOH)_7$
588.90	$HCOO(NaCOOH)_8$
656.88	$HCOO(NaCOOH)_9$
724.87	$HCOO(NaCOOH)_{10}$
792.86	$HCOO(NaCOOH)_{11}$
860.85	$HCOO(NaCOOH)_{12}$
982.83	$HCOO(NaCOOH)_{13}$
996.82	$HCOO(NaCOOH)_{14}$
1064.81	$HCOO(NaCOOH)_{15}$
1132.80	$HCOO(NaCOOH)_{16}$
1200.78	$HCOO(NaCOOH)_{17}$
1268.78	$HCOO(NaCOOH)_{18}$
1336.76	$HCOO(NaCOOH)_{19}$
1404.75	$HCOO(NaCOOH)_{20}$
1472.73	$HCOO(NaCOOH)_{21}$
1540.72	$HCOO(NaCOOH)_{22}$
1608.70	$HCOO(NaCOOH)_{23}$
1676.70	$HCOO(NaCOOH)_{24}$
1744.68	$HCOO(NaCOOH)_{25}$

Table S10. MS/MS fragment ions of small proanthocyanidin oligomers detected by TopN method in the ultrahigh-performance liquid chromatographic tandem mass spectrometric analysis.

DP	Monomeric units*	Type	Mcalculated	$[M–H]^-$	Main MS/MS fragments (*m/z*)**
1	PC		290.07904	289	109, 123, 245
1	PD		306.07396	305	109, 125, 137, 261
1	PC+G		442.07904	441	109, 125, 169, 245, 289
2	2PC	A	576.12678	575	109, 125, 161, 285, 289, 407, 423, 449
2	2PC	B	578.14243	577	109, 125, 161, 287, 289, 407, 425, 451
2	PC+PD	B	594.13734	593	109, 125, 137, 177, 287, 289, 303, 305, 407, 423, 425, 441, 467
2	2PD	B	610.13226	609	109, 125, 137, 177, 303, 305, 423, 441, 483
2	2PC+G	B	730.14243	729	109, 125, 137, 161, 169, 289, 407, 425, 441, 451, 577, 603
3	3PC	A	864.19017	863	109, 125, 161, 285, 289, 411, 451, 559, 573, 693, 711
3	3PC	B	866.20582	865	109, 125, 161, 287, 289, 405, 407, 423, 425, 449, 451, 575, 577, 695, 713, 739
3	2PC+PD	A	880.18509	879	109, 125, 137, 161, 177, 285, 305, 411, 423, 467, 559, 573, 709, 727
2	2PC+2G	B	882.16435	881	109, 125, 137, 161, 169, 289, 407, 541, 559, 577, 603, 711, 729
3	2PC+PD	B	882.20073	881	109, 125, 137, 177, 287, 289, 303, 305, 405, 407, 421***, 423, 425, 439***, 441, 465, 467, 575, 577, 591, 593, 695, 711, 713, 729, 755
3	PC+2PD	A	896.18000	895	109, 125, 137, 177, 285, 305, 411, 423, 467, 483, 559, 575, 709, 725***, 727, 743
3	PC+2PD	B	898.19565	897	109, 125, 137, 177, 287, 289, 303, 305, 405, 407, 421, 423, 425, 439, 441, 465, 467, 481, 483, 591, 593, 607, 609, 711, 727, 729, 771
3	3PD	A	912.17492	911	109, 125, 137, 177, 301, 305, 423, 427, 483, 575, 599, 725, 743
3	3PD	B	914.19057	913	109, 125, 137, 177, 303, 305, 421, 423, 439, 441, 481, 483, 607, 609, 727, 745, 787***
3	3PC+G	B	1018.20582	1017	109, 125, 137, 161, 169, 287, 289, 405, 407, 423, 441, 449, 451, 559, 575, 577, 603, 695, 729, 847, 865, 891
4	4PC	A	1152.25356	1151	109, 125, 161, 285, 289, 411, 451, 559, 573, 693, 711, 863, 981
4	4PC	B	1154.26921	1153	109, 125, 161, 287, 289, 405, 407, 423, 425, 449, 451, 575, 577, 696, 713, 739, 983, 1001, 1027

DP	Monomeric units*	Type	Mcalculated	[M–H]⁻	Main MS/MS fragments (*m/z*)**
3	3PC+2G	B	1170.22774	1169	109, 125, 137, 161, 169, 287, 289, 405, 407, 431, 433, 439, 441, 449, 451, 541, 557, 559, 575, 603, 659, 677, 695, 711, 729, 829, 847, 881, 891, 999, 1017, 1043
4	PC+3PD	B	1202.25396	1201	109, 125, 137, 177, 303, 305, 405, 421, 423, 439, 441, 465, 481, 483, 591, 593, 607, 609, 725, 727, 787, 895, 913, 1015, 1031
4	4PD	B	1218.24887	1217	109, 125, 137, 177, 303, 305, 421, 423, 439, 441, 481, 483, 607, 609, 725, 727, 745, 787, 911, 913, 1031, 1049, 1091
4	4PC+G	B	1306.26921	1305	109, 125, 137, 161, 169***, 287, 289, 405, 407, 441, 449, 451, 559, 575, 577, 603, 729, 847, 863, 891, 983, 1135, 1153
3	3PC+3G	B	1322.23870	1321	109, 125, 137, 161, 169, 287, 289, 407, 539, 541, 557, 559, 709, 711, 727, 729, 829, 881, 981, 999, 1017, 1043, 1151, 1169

* The monomeric units are not presented in the sequential order. There can exist several different isomers.
** Fragments are produced by quinone methide and direct cleavage of the interflavanoid bond and by retro-Diels–Alder fragmentation and heterocyclic ring fission. In addition, the sequential cleavage of water is often detected. The fragments with the same *m/z* values are not necessarily similar fragments. For example, the fragmentation of B-type procyanidin trimer (866 Da) produces a fragment at *m/z* 575 which is the quinone methide product corresponding for the cleavage of the lower interflavonoid bond but has the very same integer *m/z* value as the A-type procyanidin dimer (576 Da) having the additional ether linkage.
*** Minor fragment but it supports the patterns obtained for similar proanthocyanidins and is therefore reported.
